# Neurosteroid withdrawal disrupts GABAergic system development in human cortical organoids: implications for preterm birth

**DOI:** 10.3389/fncel.2025.1715823

**Published:** 2025-12-17

**Authors:** Helene Lacaille, Ekaterina Lebayle, Barbara Corneo, Claire-Marie Vacher, Anna A. Penn

**Affiliations:** 1 Division of Neonatology, Department of Pediatrics, Vagelos College of Physicians and Surgeons and New York-Presbyterian Morgan Stanley Children’s Hospital, Columbia University, New York, NY, United States; 2 Columbia Stem Cell Initiative, Stem Cell Core, Columbia University Irving Medical Center, New York, NY, United States

**Keywords:** organoids, cerebral cortex, placenta, brain development, neurosteroid, allopregnanolone, interneuron, GABA

## Abstract

Preterm birth substantially elevates the risk of neurological and cognitive disorders. Recent evidence suggests that the abrupt loss of placental support, particularly the cessation of neurotrophic and neuroprotective hormones, alters neurodevelopmental trajectories and may contribute to neurodevelopmental risk associated with prematurity. Our study investigates how the placental steroid hormone, allopregnanolone (ALLO), affects cerebral cortex development using human cortical organoid models. Our findings reveal that while ALLO exposure produces modest effects on overall cortical development, its withdrawal specifically disrupts GABAergic but not glutamatergic neuronal development. These results demonstrate that placental hormones, including ALLO, may target specific neuronal populations critical for cortical function, identifying potential therapeutic interventions following placental loss in human preterm neonates.

## Introduction

Preterm birth and placental conditions are linked to abnormal brain development (Kratimenos and Penn, 2019; Vacher et al., 2023, 2025; du Plessis and Volpe, 2025). A 2023 report revealed that in 2020, an estimated 13.4 million babies lost placental support upon premature birth (before 37 gestational weeks), 15% of which occurred at less than 32 weeks of gestation, requiring more neonatal care (Ohuma et al., 2023). This can lead to lifelong neurological and psychiatric disorders, including autism, epilepsy, and cognitive impairments (Hee Chung et al., 2020; Song, 2023). As preterm care has improved, cystic areas of brain injury have become less common but cortical dysmaturation has emerged as a significant contributor to neurobehavioral impairments in childhood (Back and Miller, 2014; Dean et al., 2014; Volpe, 2019). Indeed, preterm infants show persistent structural and functional connectivity abnormalities, which are increasingly linked to neurodevelopmental impairments (Rogers et al., 2018). Recent work has further shown that preterm birth is associated with atypical dynamic functional connectivity in the neonatal brain, which correlates with altered social, sensory, and repetitive behaviors in early childhood (França et al., 2024), highlighting the lasting impact of early disruptions in brain network development.

Research has traditionally focused on *in-utero* or perinatal injuries (hypoxia, maternal immune activation, hyperoxia, and hypoxic–ischemic encephalopathy). However, the loss of placental factors may be equally critical as acquired injuries and could inform the development of therapeutic strategies to protect preterm brain development. Although human and preclinical studies suggest pregnancy hormones influence cognitive development (Reis and Petraglia, 2001; Pasca and Penn, 2010; Vacher et al., 2025), few have examined the direct effects of alterations in placentally produced hormones. Placental ALLO (Gilbert Evans et al., 2005; Vacher et al., 2021) crosses the blood–brain barrier and acts on neural cells primarily as a positive allosteric modulator of GABA-A receptor activity (Legesse et al., 2023), regulating cell proliferation, migration, survival, and process outgrowth (Charalampopoulos et al., 2004; Wang et al., 2005; Shimizu et al., 2015; Zamora-Sánchez et al., 2022). Placental ALLO production, and consequently fetal brain levels, peak during mid-to-late gestation, coinciding with both cerebral cortex neuronal maturation (Kelleher et al., 2011; Vacher et al., 2021) and preterm birth. This timing suggests a potential role of acute ALLO withdrawal in preterm brain injuries and highlights opportunities for hormone replacement therapies. Using a mouse model of placental ALLO insufficiency (the plKO mouse model) (Vacher et al., 2021), we previously showed that lack of placental ALLO results in impaired cerebellar myelination and an autism spectrum disorder (ASD)-like phenotype in male mice.

Here, we hypothesize that placental ALLO withdrawal contributes to the cortical dysmaturation in preterm survivors. To test this, we used cortical organoids derived from human induced pluripotent stem cells (hiPSCs) to assess the direct influence of ALLO exposure and withdrawal on distinct cortical progenitor cells. These organoids serve as a simplified *in vitro* model that replicates many structural and functional characteristics of the developing human cortex (Arlotta and Paşca, 2019; Kelley and Pașca, 2022). We further patterned them into dorsal (DCOs) and ventral (VCOs) cortical organoids to examine the cortical glutamatergic and GABAergic system maturation, respectively (Birey et al., 2017; Sloan et al., 2018). We show that ALLO withdrawal disrupts the development of the GABAergic system and the progression of interneuron progenitor lineage. These findings validate the critical role and direct influence of placental hormones in cortical development and inform future therapeutic strategies for preterm birth-related complications.

## Materials and methods

### Cortical organoids

#### Procedure

Human brain organoids were generated as previously described (Birey et al., 2017; Sloan et al., 2018) using a human induced pluripotent stem cell line (hiPSC) FA0000010 (CUIMCi001-A), derived from a healthy male donor (Patel et al., 2020). The cell line was obtained from Columbia University Irving Medical Center and is available through RUCDR Infinite Biologics.^1^ Briefly, to generate dorsal cortical organoids (DCOs), hiPSC-derived spheroids were patterned by double SMAD inhibition (Dorsomorphin and SB431542; Tocris, 3,093 and 1,614), for 6 days from DIV (day *in vitro*) 0 to DIV6, then cultured in presence of FGF2 and EGF (RD Systems, 233-FB and 236-EG) for 11 days (DIV7 to DIV25). Ventral cortical organoids (VCOs) were patterned by double SMAD inhibition supplemented by the WNT inhibitor IWP2 (Selleck Chemicals, S7085) for 6 days then cultured in presence of FGF2 and EGF with IWP2 and the SHH inhibitor SAG (Tocris, 4,366) for 11 days. Both DCOs and VCOs were cultured in medium supplemented with BDNF (R&D Systems, 248-BDB) and NT3 (PeproTech, 450–03) for another 18 days, then without growth factors from day 46 onward.

#### Treatment

DCOs and VCOs were either treated with 125 nM ALLO (Tocris, 3,653) diluted in DMSO (Millipore Sigma, D4540), or with DMSO (equivalent 0.05%) from DIV28 to DIV67. At nanomolar concentration, ALLO has neurotrophic properties, promoting neuronal proliferation (Wang et al., 2005), neuronal differentiation (Chen et al., 2020) and maturation (Wang et al., 2023). A concentration of 100–150 nM is comparable to physiological pregnancy levels of ALLO during pregnancy in rats (Concas et al., 1998), and human (Luisi et al., 2000).

#### Collection

Cortical organoids were collected at DIV26, 55, 78, 95, and stored in TRIzol™ (ThermoFisher Scientific, 15,596,018) for RNA extraction. At DIV138, organoids were either collected for RNA extraction or stored in PFA (ThermoFisher Scientific, J19943. K2) for immunohistochemistry.

### Real-time PCR (RT-PCR)

Organoids were homogenized in TRIzol™ Reagent; total RNA was extracted with the RNeasy Mini Kit (Qiagen, 74,104) and quantified with a NanoDropND-2000C (Thermo Fisher Scientific). A total of 300 ng of RNA was converted to cDNA using iScript cDNA Synthesis Kit (Bio-Rad, 1,708,891). All primer pairs were validated in-house for efficiency and specificity (Supplementary Table S1). RT-PCR experiments were performed on cDNA samples with SsoAdvanced Universal SYBR Green Supermix (Bio-Rad, 1,725,271) and specific primers at 100 nM using the ABI Prism 7,500 Sequence Detection System (Thermo Fisher) for 40 cycles. The cDNA-generated signals for target genes were normalized to the glyceraldehyde-3-phosphate dehydrogenase (*GAPDH*) housekeeping gene. Relative gene expression was calculated using the 2^−∆∆Cq^ method. Results are expressed as fold change (FC) relative to undifferentiated hiPSCs levels.

#### Calculation of the segmented developmental index

The index was derived from the ratio of averaged expression of significantly up-regulated genes to averaged expression of significantly down-regulated genes, as previously described (Gandal et al., 2012; Lacaille et al., 2021). Details of the calculation can be found in Supplementary Figure S1. Briefly, the expression of 23 transcripts related to neuronal development, was assessed across four developmental segments: DIV26-DIV55, DIV55-DIV78, DIV78-DIV95, and DIV95-DIV138. For each segment, Pearson correlation coefficients were calculated for each gene within the DMSO group. Genes were classified as significantly up- or down-regulated based on the sign of their correlation coefficient and a *p* < 0.05. All genes were given equal weight in the calculation. Then, the ratio of averaged up-regulated to averaged down-regulated gene expression served as a developmental index. The same set of genes was then assessed in the corresponding ALLO group, and the index was calculated using the same methodology. To quantify the developmental index’s trajectory, linear regression slopes were fitted to measure developmental changes across these segments and compared between treatment with an ANCOVA.

#### Clustering

To cluster the genes according to their developmental trajectory the maSigPro R package was used (Nueda et al., 2014).

### Immunohistochemistry

#### Tissue preparation

Organoids were fixed in 4% PFA, cryoprotected in a 20% sucrose solution, then embedded in a 1:1 sucrose-Tissue-Tek^®^ O. C. T. Compound (Sakura Finetek, 4,583). Blocks were cut into 10-μm-thick sections with a cryostat. Frozen sections were allowed to equilibrate to room temperature for 1 h before staining.

#### Procedure

Tissue sections were rinsed in PBS-Triton 0.3% (PBS-T) then blocked in PBS-T with 10% normal donkey serum (NDS), followed by overnight incubation at 4 °C in PBS-T-10% NDS with the following primary antibodies: Beta-3 tubulin (B3TUB; 1:500, Cell Signaling, D71G9), Calretinin (CALB2; 1:1000, Millipore Sigma, AB1550), Doublecortin (DCX; 1:500, Abcam, ab113435), GABA transporter type 1 (GAT1; 1:200, AbClonal, A15099), GABA transporter type 3 (GAT3; 1:100, Santa Cruz Biotechnology, sc-376001), Glutamate decarboxylase 65–67 (GAD65-67; 1:100, Santa Cruz Biotechnology, sc-365180), Glial fibrillary acidic protein (GFAP; 1:1000, Dako, Z0334), HuC/HuD (HU; 1:500, Invitrogen, A-21271), Ki67 (MKI67; 1:500, Abcam, ab156956), Nestin (NES; 1:250, Novus Biologicals, NB100-1604), NeuN (NEUN; 1:500, Abcam, ab177487), Somatostatin (SST; 1:300, Santa Cruz Biotechnology, sc-7819), SRY-box 2 (1:500, SOX2; Millipore, AB5603), Vesicular GABA transporter (VGAT; 1:300, AbClonal, A3129), Vimentin (VIM; 1:500, Santa Cruz Biotechnology, sc-373717). For secondary detection, appropriately matched Alexa Fluor-conjugated secondary antibodies (1:500, ThermoFisher) were incubated for 90 min in PBS-T at room temperature. Then, sections were incubated with DAPI, mounted with Fluoromount G (ThermoFisher, 0100–01) on a glass coverslip before epifluorescence examination at 20x (ZEISS Axio Imager 2). Images were tiled (Stereo Investigator, MBF Bioscience).

#### Quantification

Cell counting was performed using QuPath (version 0.4.3) automated quantification for fluorescently labeled cells (Courtney et al., 2022). Briefly, DAPI-based detection was used to identify cell nuclei. The object classifier was trained independently for each marker and applied to all tissue sections for automatic detection (Supplementary Figure S2). The results were reviewed for accuracy, and cell density was normalized as percentage of DAPI+ cells.

### Statistics

All experiments and analyses were performed blind to conditions. Statistical analyses were performed using R. Slope differences for the developmental index were compared with an ANCOVA. When assessing groups with two or more variables, data were analyzed using ANOVA with Benjamini-Hochberg correction for multiple comparisons. General effects of treatment and day were assessed using ANOVA interaction effects (denoted by ^#^), and *post hoc* comparisons are indicated with asterisks (*). The null hypothesis was rejected for *p* < 0.05.

## Results

To test the direct effects of ALLO exposure and withdrawal on human cortical development, independent of other systemic factors, we used cortical organoids. Ventral and dorsal cortical organoids (VCOs and DCOs, respectively) were cultured independently for up to 138 days (DIV138). Each type of cortical organoid was designed to examine distinct neuronal populations: GABAergic cortical interneurons in VCOs and glutamatergic principal neurons in DCOs. After patterning, organoids were exposed to either ALLO or DMSO during the maturation phase (DIV28-67).

### Cortical organoids express the GABA-A receptor subunits required for ALLO action

**Figure 1:**
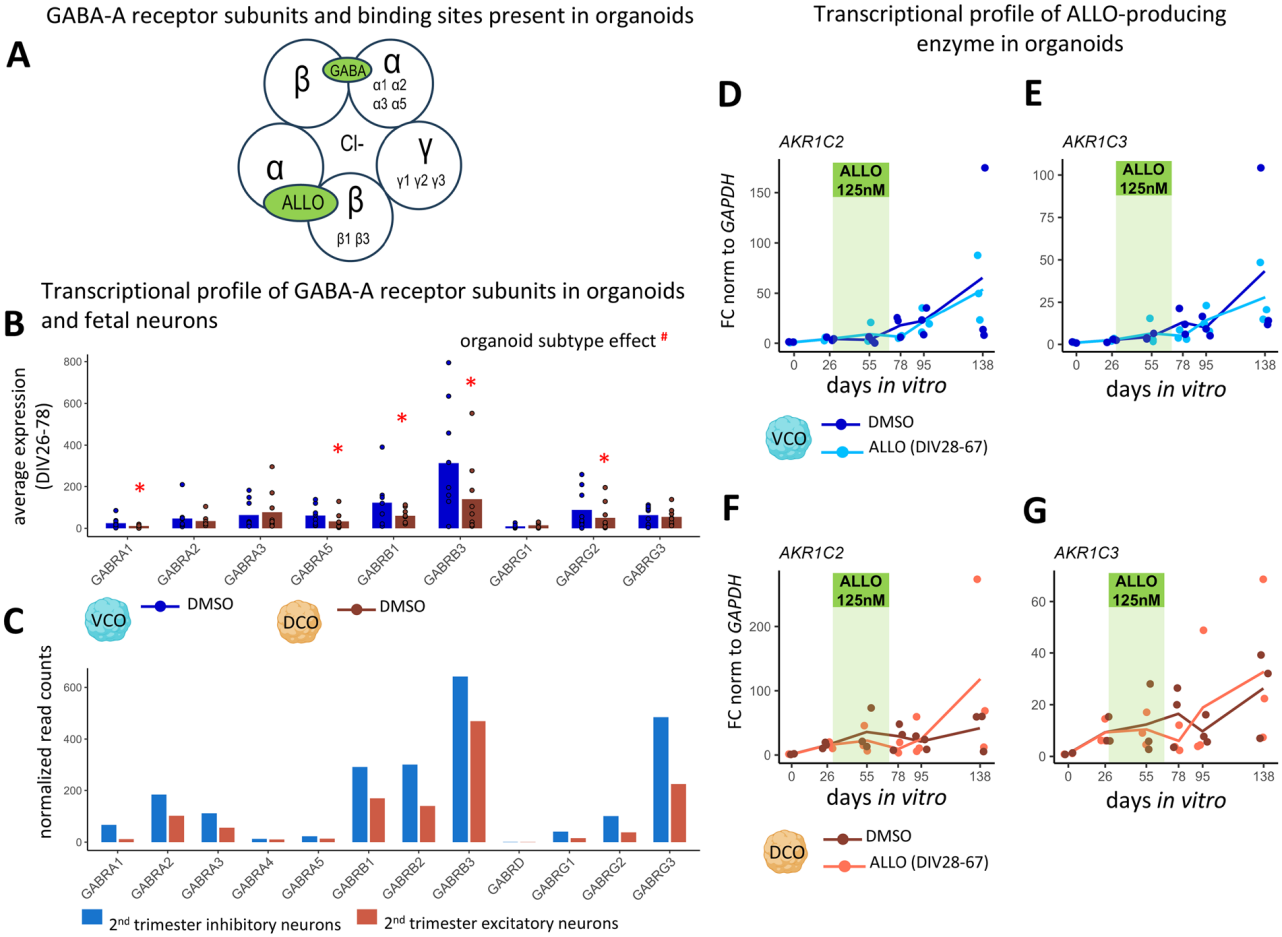
Cortical organoids as a model to study ALLO exposure and withdrawal **(A–C)** GABA-A receptor subunit gene expression is enriched in interneuron populations compared to principal cells. **(A)** The GABA-A receptor subunits present in both organoid subtypes allow GABA and neurosteroid binding (ALLO). **(B)** Average expression of GABA-A receptor subunits, in VCOs (ventral cortical organoids, blue) and DCOs (dorsal cortical organoids, brown) treated with DMSO, during the period of ALLO treatment (DIV28-DIV67), expressed as scaled relative abundance to *GAPDH* expression, and multiplied by 10,000. *n* = 9. Two-way ANOVAs with BH correction compared organoid subtypes and subunits. #organoid subtype effect. Multiple comparisons: *p < 0.05. **(C)** Transcriptional expression of GABA-A subunits in human fetal tissues between gestational weeks 13 and 17, after re-analysis of GSE156793 for cerebrum inhibitory and excitatory neurons, expressed as average of normalized read counts. **(D–G)** ALLO does not modulate AKR1Cs expression in cortical organoids. Transcriptional profile of ALLO-producing enzyme, in **(D,E)** VCOs (blue) and **(F,G)** DCOs (orange) treated with DMSO (dark) or ALLO (125 nM, light; from DIV28 to 67, highlighted in green). **(D,F)** *AKR1C2* (Aldo-keto reductase family 1 member C2) and **(E,G)** *AKR1C3* (Aldo-keto reductase family 1 member C3) at each time point (DIV0, 26, 55, 78, 95, 138). *n* = 3. Two-way ANOVAs with BH correction compared treatments and days.

We first tested whether organoids express the embryonic GABA-A receptor subunits necessary for ALLO’s action (Kilb et al., 2013). ALLO’s allosteric action on GABA-A receptors requires its binding at the interface between the α and β subunits of the pentameric receptor, specifically receptors containing α2-5 assembled with β3 and γ2 (Hosie et al., 2009; Legesse et al., 2023; Figure 1A). Although not the primary binding site, the δ subunit confers enhanced sensitivity and mediates the long-lasting effects of neurosteroids (Smith et al., 2007). We assessed GABA-A receptor subunit gene expression in non-ALLO-treated organoids between DIV26 and DIV78, covering the ALLO exposure period in treated organoids (Figure 1B). Of all 19 subunits tested, both organoid types expressed GABA-A receptor subunits α1-3, α5, β1, β3, and γ1-3. The remaining subunit transcripts were not detectable. This expression profile supports the organoids’ capacity to form functional receptors with both GABA and neurosteroid binding sites, as these receptors are typically assembled from five subunits, commonly two α, two β, and one γ or δ. VCOs showed higher expression of specific subunits (α1, α5, β1, β3, γ2) than DCOs, consistent with our re-analysis of published second-trimester transcriptomic data (GSE156793) (Cao et al., 2020) showing higher GABA-A subunit gene expression in interneuron progenitors than in pyramidal progenitors (Figure 1C). Of the 11 GABA-A receptor subunits expressed during the second trimester in fetal neurons (Figure 1C), nine of these were observed in our organoid model (Figure 1B). The δ subunit was absent in both fetal neurons and organoids, consistent with its known developmental trajectory. Notably, the β3 subunit was the most highly expressed in both datasets (Figures 1B,C). Overall, the main subunits expressed and those not expressed were consistent between fetal tissue and organoids, supporting the developmental relevance of our organoid system. We also examined whether ALLO treatments modified the expression of ALLO synthesis enzymes, as this could potentially bias results through changes in endogenous ALLO production. Both key synthetic enzyme transcripts, *AKR1C2* (the main alpha-hydroxysteroid dehydrogenase enzyme in humans for ALLO production) and *AKR1C3* (an alternative synthesis enzyme) (Penning et al., 2000), are expressed in organoids, and their gene expressions remained unchanged with ALLO exposure (Figures 1D–G). This indicates that any observed effects on neuronal development would not be related to changes in the endogenous production of ALLO in the cortical organoids.

### ALLO withdrawal delays the development of ventral cortical organoids

We investigated the effects of ALLO exposure and withdrawal on organoid developmental progression by analyzing time-dependent expression of genes linked to neural cell development (Supplementary Figures S3, S4). We selected genes of interest based on the literature, including markers for neuroepithelial cells (*SOX10, SOX2, PCNA, NES*), radial glia (*VIM*, *GFAP*, *NCAM1, SLC1A3*), markers associated with region-specific progenitor and early neuronal identities (*FOXG1*, *ASCL1*, *TTF1*, *NR2F2* for VCOs; and *FOXG1*, *SATB2*, *FOXP2*, *PAX6* for DCOs), immature neurons (*DCX*, *TUBB3*), mature neurons (*RBFOX3*, *MAP2*, *ENO2*, *SYP*, *NEFM*, *NEFL*, *DLG4*), cortical interneurons (*CALB2*, *GAD1*, *GAD2*, *SST*, *PVALB*, for VCOs) and pyramidal cells (*CUX1*, *SATB2*, *SLC17A7*) (Paşca et al., 2015; Birey et al., 2017; Sloan et al., 2018). First, we examined individual gene expression profiles. In VCOs, only *FOXG1*, critical for the timing and production of GABAergic neurons (Miyoshi et al., 2021), was acutely upregulated by ALLO exposure at DIV55 (Supplementary Figure S3). Following ALLO withdrawal, expression of *RBFOX3* (NeuN) and *MAP2* (involved in dendritic development) increased, while expression of *DCX* and axonal formation genes *NEFM* and *NEFL* decreased (Supplementary Figure S3), suggesting alterations in neuronal arborization and circuit formation. In DCOs, ALLO treatment reduced *VIM* expression. After ALLO withdrawal, expression of *CUX1* and *PAX6* (both involved in cortical pyramidal cell specification) and *DCX* decreased, while *ENO2* (neuron-specific enolase expressed in mature neurons) expression increased (Supplementary Figure S4). These results suggest that in DCOs ALLO promotes and maintains cortical neuron identity, and that its withdrawal causes a shift toward neuronal maturation.

**Figure 2:**
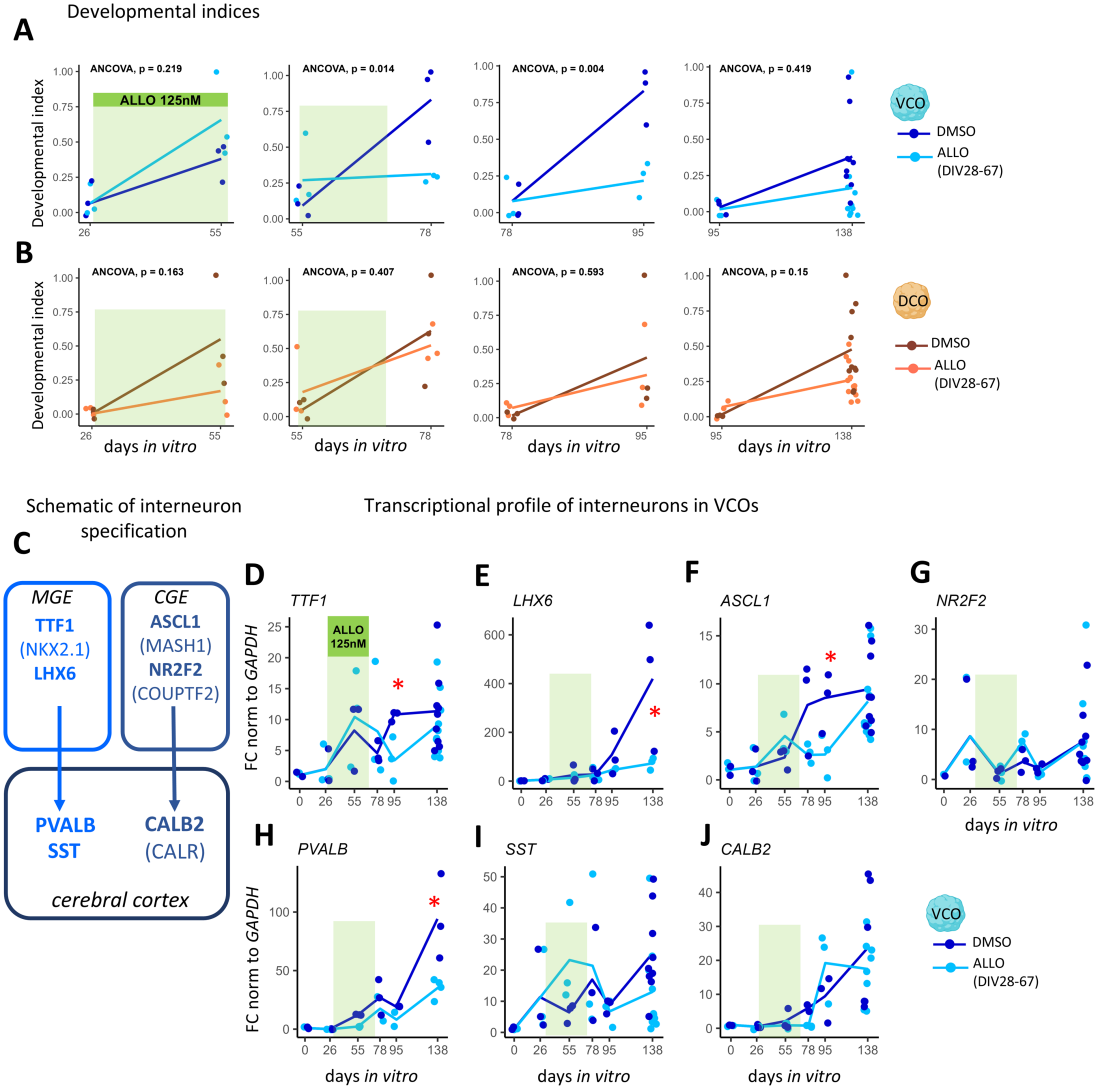
ALLO exposure and ALLO withdrawal alter the development of VCOs. **(A,B)** Segmented developmental index based on the average of up versus down-regulated genes, in **(A)** VCOs (ventral cortical organoids, blue) and **(B)** DCOs (dorsal cortical organoids, orange) treated with DMSO (dark) or ALLO (125 nM, light, from DIV28 to 67, highlighted in green), at each segment, DIV (days *in vitro*) 26-DIV55, DIV55-DIV78, DIV78-DIV95, and DIV95-DIV138. ANCOVA compared slope differences. **(C)** Simplified schematic of the embryonic and mature fate of the cortical interneuron markers in the MGE (medial) and CGE (caudal ganglionic eminence) analyzed in this study. **(D–J)** Transcriptional profiles of interneuron-related transcripts in VCOs (ventral cortical organoids) treated with DMSO (dark) or ALLO (light) at each time point (DIV0, 26, 55, 78, 95, 138). **(D–G)** Interneuron progenitors and **(H–J)** mature interneuron subtypes. **(D)** *TTF1* (Thyroid Transcription Factor 1). **(E)** *LHX*6 (LIM homeobox 6). **(F)** *ASCL1* (Achaete-Scute Family BHLH Transcription Factor 1). **(G)** *NR2F2* (Nuclear Receptor Subfamily 2 Group F Member 2). **(H)** *PVALB* (Parvalbumin). **(I)** *SST* (Somatostatin). **(J)** *CALB2* (Calbindin 2). DIV0-DIV95 *n* = 3, DIV138 *n* = 3–9. Two-way ANOVAs with BH correction compared treatments and days. Multiple comparisons: *p < 0.05.

To compare how ALLO exposure and withdrawal affected the overall developmental progression of VCOs and DCOs, we calculated time-segmented developmental indices (see Methods; Lacaille et al., 2021; Figures 2A,B; Supplementary Figure S1 and Supplementary Table S2). While ALLO exposure did not significantly alter VCOs’ developmental trajectory (DIV26-DIV55), its removal caused an immediate and prolonged developmental delay (DIV55-DIV78; *p* < 0.05) (Figure 2A) that persisted until DIV95 (DIV78-DIV95; *p* < 0.005) (Figure 2A). By DIV138, the developmental slope of VCOs matched that of untreated controls (Figure 2A). This developmental delay was marked by a short and long term downregulation of neuronal lineage genes. The short-term effect of ALLO withdrawal at DIV78 (Cluster 1) (Supplementary Figure S5) affected neuronal development broadly, including genes involved in neurogenesis and early neuronal differentiation (*SOX2*, *TUBB3*, *GFAP*), neuronal maturation (*MAP2*, *RBFOX3*), functional specialization (*CALB2*, *PVALB*), and synaptic formation (*SYP*). The long-term effect at DIV95 (Cluster 2) (Supplementary Figure S5) impacted genes involved in interneuron progenitor progression (*DCX*, *ASCL1*, *NEFL*, *TTF1*). In contrast, DCO developmental trajectory remained unaffected by either ALLO exposure or withdrawal (Figure 2B).

Since ALLO treatments primarily altered developmental trajectories in VCOs (which model the ganglionic eminences that generate GABAergic interneurons), we investigated effects on interneuron lineage progression. We analyzed transcripts associated with the medial (*TTF1, LHX6*) and caudal (*ASCL1*, *NRF2*) ganglionic eminences and their resulting cortical interneurons (*PVALB*, *SST*, and *CALB2,* respectively) (Figure 2C). While ALLO exposure itself (DIV55) did not affect interneuron lineage marker gene expression (Figures 2D–J), its withdrawal led to significant downregulation of key interneuron progenitor and specification markers: *TTF1* (DIV95; −68%, *p* < 0.05) (Figure 2D), *LHX6* (DIV138; −72%, *p* < 0.05); (Figure 2E) and, *ASCL1* (DIV95; −68%, *p* < 0.05) (Figure 2F) as well as the cortical interneuron marker *PVALB* (DIV138; −62%, *p* < 0.05) (Figure 2H). The downregulation of *TTF1*, *LHX6*, and *PVALB* supports a specific impact on the medial ganglionic eminence progenitors.

### ALLO withdrawal alters the development of cortical interneurons

**Figure 3:**
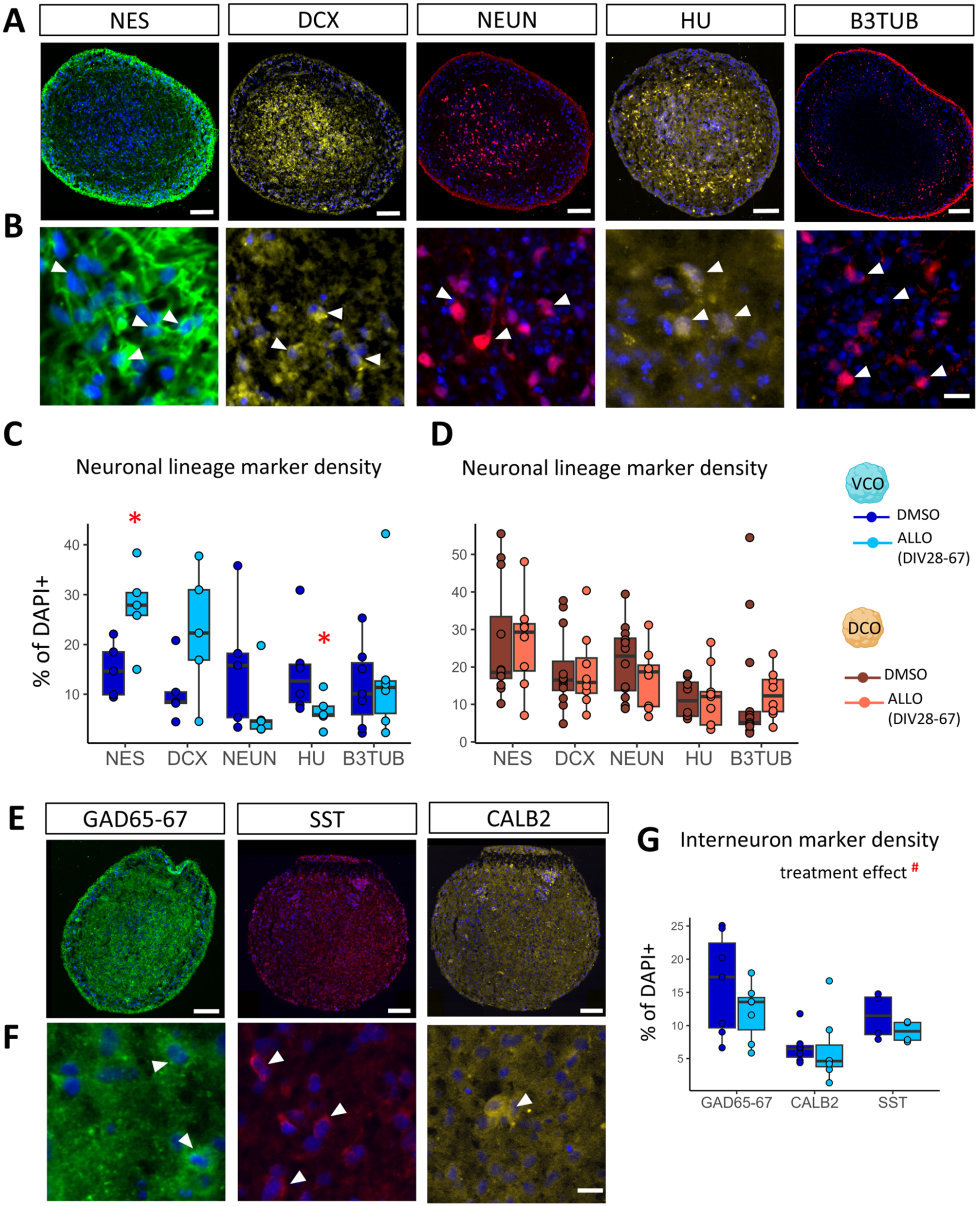
ALLO delays the expression of the GABAergic neuronal lineage. **(A–D)** Cumulative effect of ALLO exposure and withdrawal at DIV138 on markers of neuronal lineage. **(A)** Representative illustration of whole organoid staining, scale = 100 μm. **(B)** Representative high magnification illustrations of positive cells, scale = 10 μm, arrows. **(C,D)** Quantification in **(C)** VCOs (ventral cortical organoids, blue) and **(D)** DCOs (dorsal cortical organoids, orange) treated with DMSO (dark) or ALLO (125 nM, light, from DIV28 to 67), normalized as percentage of DAPI+ cells. NES, nestin; DCX, Doublecortin; NEUN; HU; and B3TUB, Beta III Tubulin. **(E–G)** Cumulative effect of ALLO exposure and withdrawal at DIV138 on interneuron markers in VCOs. **(E)** Representative illustration of whole organoid staining, scale = 100 μm. **(F)** Representative high magnification illustrations of positive cells, scale = 10 μm, arrows. **(G)** Quantification in VCOs treated with DMSO (dark) or ALLO (light), normalized as percentage of DAPI+ cells. GAD65-67, Glutamate Decarboxylase 65–67; CALB2, Calretinin; SST, Somatostatin. *n* = 5–9. Two-way ANOVAs with BH correction compared treatments and markers. Multiple comparisons: *p < 0.05.

Histological analysis (Figures 3A–D; Supplementary Figure S6) of VCOs at DIV138 (approximating late fetal stages) (Gordon et al., 2021) complemented these transcriptional findings, revealing increased densities of immature neurons (NES: +84%, *p* < 0.05; DCX: +115%, trend with *p* = 0.066) (Figure 3C) and decreased densities of mature neurons (NEUN: −55%, *p* = 0.12; HU: −54%, *p* < 0.01) (Figure 3C). This indicates a long-term impact of ALLO exposure and withdrawal on neuronal maturation, most likely affecting the interneuron population that VCOs were designed to produce. This effect was specific to neuronal development, as proliferation and glial differentiation remained unchanged in VCOs (Supplementary Figure S7). We therefore focused on interneurons, which VCOs are designed to produce, and found a long-term reduction in the density of mature interneurons (immunostained for GAD65-67, CALB2, or SST) at DIV138 (treatment effect, *p* < 0.05) (Figures 3E–G; Supplementary Figure S8). DCOs only showed decreased VIM-positive cells (−33%, *p* < 0.05) (Supplementary Figure S7), suggesting ALLO altered the regulation of neuronal progenitors or radial glia development.

### ALLO loss alters the developmental trajectory of cortical GABAergic signaling components

**Figure 4:**
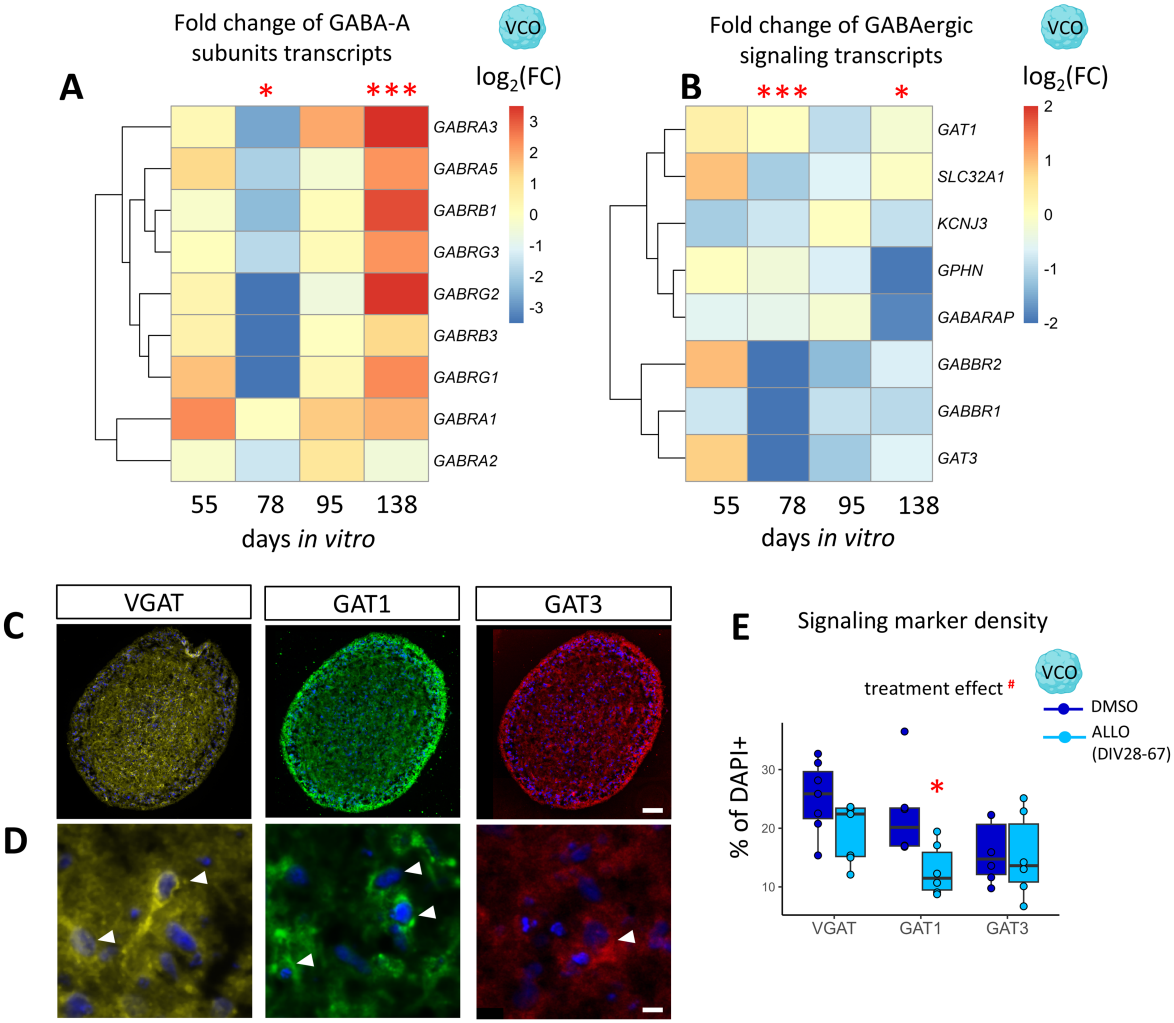
ALLO withdrawal downregulates elements of the GABAergic system in VCOs. **(A)** Heatmap representing the fold change of GABA-A receptor subunits transcripts of ALLO-exposed organoids (125 nM; from DIV28 to 67) over DMSO-exposed organoids at each time point (DIV55, 78, 95, 138) in VCOs (ventral cortical organoids), ###treatment effect, ###treatment:day effect. **(B)** Heatmap representing the fold change of eight transcripts involved in GABAergic signaling of ALLO-exposed over DMSO-exposed VCOs at each time point (DIV55, 78, 95, 138), #treatment:day. *n* = 3. Two-way ANOVAs with BH correction compared treatments and days. Multiple comparisons: *p < 0.05, ***p < 0.001. **(C–E)** Cumulative effect of ALLO exposure and withdrawal at DIV138 on markers of GABAergic signaling. **(C)** Representative illustration of whole organoid staining, scale = 100 μm. **(D)** Representative high magnification illustrations of positive cells, scale = 10 μm, arrows. **(E)** Quantification in VCOs treated with DMSO (dark) or ALLO (light), normalized as percentage of DAPI+ cells. #treatment effect. VGAT, Vesicular GABA transporter; GAT1, GABA transporter 1; GAT3, GABA transporter 3. *n* = 5–6. Two-way ANOVAs with BH correction compared treatments and markers. Multiple comparisons: *p < 0.05.

We next examined the effects of ALLO exposure and withdrawal on GABAergic signaling elements in VCOs. Consistent with our observations of interneuron lineage markers, ALLO exposure did not significantly alter GABAergic signaling gene expression at DIV55 (Figures 4A,B). However, after ALLO was removed, a complex dynamic response emerged. At DIV78, we observed an overall downregulation of GABA-A receptor subunits (Figure 4A), with a notable decrease in *GABRB3* (DIV78; −80%, *p* < 0.05) (Supplementary Figure S9A). *GABRB3* is a subunit crucial for ALLO binding, and its reduced expression is linked to receptor desensitization (Sugasawa et al., 2020). At DIV138, several GABA-A receptor subunits, specifically *GABRA1, GABRA5*, *GABRB1*, *GABRB3*, and *GABRG2*, were overexpressed (Supplementary Figure S9A), suggesting long-term dysregulation of GABA-A receptor developmental expression with possible receptor subunit rearrangement. These receptors mediate both synaptic GABA responses present in VCOs (Birey et al., 2017) and non-synaptic GABA responses that regulate interneuron proliferation, migration, and differentiation (Kilb et al., 2013). Initial receptor reduction may impair the organoids’ ability to sense ambient GABA, while later overexpression at DIV138 suggests a long-term dysregulation of GABA signaling after ALLO removal. ALLO withdrawal also led to overall downregulation of key GABAergic signaling genes, including GABAergic transporters (*SLC32A1, SLC6A1, SLC6A11*) and postsynaptic elements (*KCNJ3*, *GPHN*, *GABARAP*, *GABBR2*, *GABBR1*) (DIV78, *p* < 0.001) (Figure 4B). The downregulation persisted at DIV138 with a significant treatment effect observed (*p* < 0.05; Figure 4B), although only *GPHN* and *GABARAP* remained individually significant at this timepoint (Supplementary Figure S9B). Immunohistochemistry at DIV138 confirmed a sustained reduction of signaling marker levels (i.e., VGAT, GAT1, GAT3; treatment effect, *p* < 0.05; GAT1: −42%, *p* < 0.05) (Figures 4C–E), suggesting less mature neurons since the expression of these markers normally increases over time.

## Discussion

### Cortical subdomain-specific effects of ALLO

Cortical organoids offer a unique platform to investigate ALLO’s direct effects, withdrawal consequences, and cumulative impact on human cortical development. While exposure of human brain organoids to ALLO exposure alone produced minimal effects, its withdrawal impaired cortical maturation, particularly within the GABAergic system, a finding with important implications for neurodevelopmental disorders. Here we showed that transient ALLO exposure impacted the developmental trajectory of VCOs (i.e., interneurons-producing-organoids) but not DCOs (i.e., principal-cells-producing-organoids), demonstrating cortical subdomain specificity in steroid allosteric effects. This difference between VCOs and DCOs may stem from both higher VCO GABA concentration, due to the presence of interneurons, and higher density of GABA-A receptors in VCOs, confirmed in our model and through the analysis of published fetal brain datasets (Cao et al., 2020). Additionally, the earlier onset of GABA-A receptor subunit expression in VCOs (DIV55 vs. DCOs at DIV78) suggests a window of susceptibility that likely amplified ALLO’s impact. While we propose that the effects we observed are mediated by ALLO’s allosteric action on GABA-A receptors, we acknowledge that neurosteroids like ALLO also act on various molecular targets beyond these receptors (Kumar et al., 2025).

### ALLO’S role in GABAergic system development

ALLO primarily altered GABAergic system development, supporting our hypothesis that it would change interneuron development both acutely and long-term. Previous research reported that subpallial spheroids (equivalent to VCOs) show no alterations in GABAergic neuron development after ALLO exposure (Birey et al., 2017), but importantly, the conditions of ALLO exposure differed in timing, duration, and concentration. ALLO exhibits concentration-dependent effects at GABA-A receptors (Puia et al., 1990; Hosie et al., 2006, 2009; Zheleznova et al., 2009; Chang et al., 2010), promoting neural progenitor proliferation at nanomolar levels through allosteric modulation, and affecting neurogenesis and survival at micromolar concentrations through direct binding to the agonist site (Gago et al., 2004; Wang et al., 2005). Treating VCOs with 125 nM ALLO revealed a complex gene regulation pattern. ALLO directly upregulated *FOXG1*, a key regulator of interneuron specification and GABAergic circuit formation. Dynamic expression of FOXG1 functions as a developmental clock for interneuron precursors, influencing their identity, migration, and final cortical allocation in a dose-dependent manner (Mariani et al., 2015; Miyoshi et al., 2021, 2024). Then following ALLO exposure and subsequent withdrawal, the ganglionic eminence interneuron progenitor genes *TTF1*, *LHX6*, and *ASCL1* were downregulated, suggesting that these genes are acutely sensitive to changes in neurosteroid levels, consistent with ALLO’s known neurotrophic effects (Bakalar et al., 2022; Hernandez and Brinton, 2022). Given that parvalbumin protein is absent during early cortical development (de Lecea et al., 1995), we interpret the decrease in *PVALB* gene expression as an early marker of lineage commitment, reflecting the delayed maturation of this interneuron subtype. Mechanistically, in immature neurons, ALLO’s positive modulation of GABA-A receptors causes chloride efflux that depolarizes the cell membrane, subsequently opening L-type calcium channels and leading to calcium influx (Wang et al., 2023). The rise in intracellular calcium activates a CREB/ELK1/c-Jun signaling pathway via CaMKII and ERK1/2, known regulator of neuronal development (Landeira et al., 2018).

### Long-term impact of transient ALLO exposure

We hypothesize that ALLO has a lasting, programming effect on gene expression that extends beyond the period of exposure. This is evidenced by the persistent downregulation of GABA signaling-related genes, and is supported by recent findings on epigenetic barriers in neural progenitors that control neuronal maturation (Ciceri et al., 2024). Indeed, at the end of our culture experiments (DIV138, i.e., late fetal stages), we observed an increased density of immature neurons (NES, DCX) and a decreased density of mature neurons (HU, NeuN, CALB2, SST, GAD65-67) in VCOs. Interestingly, this cellular phenotype contrasts with the elevated expression of mature neuronal genes (*RBFOX3, MAP2*), suggesting a disconnect between gene and protein expression. This discrepancy may be due to factors such as differential translation, protein degradation, or protein-level buffering (Buccitelli and Selbach, 2020). Additionally, the transcriptional changes observed following ALLO withdrawal (i.e., downregulation of proliferation and migration markers alongside upregulation of synaptic and maturation markers) may initially appear contradictory. However, these changes likely reflect premature developmental progression rather than accelerated healthy maturation. Neuroactive steroid signaling plays critical roles in maintaining progenitor cell proliferation and supporting neuronal migration. Withdrawal of this signaling may trigger premature exit from these developmental programs, forcing cells into post-mitotic states before completing normal developmental processes (Bakalar et al., 2022). This precocious differentiation can result in neurons with immature morphologies, reduced connectivity, and altered functional properties. Similarly, premature birth disrupts the normal timing of brain development, as shown in human studies reporting accelerated maturation of sensory and inhibitory circuits (Witteveen et al., 2023), altered dynamic functional connectivity linked to later behavioral outcomes (França et al., 2024), and stress-induced reorganization of large-scale brain networks (Lammertink et al., 2022). Our findings support a model where neuroactive steroid withdrawal disrupts the temporal coordination of developmental processes.

### Relevance to human gestation

Our organoid cultures show a developmental trajectory consistent with mid-to-late fetal cortical maturation. We observed a steep decline in the proliferation marker *PCNA* over time, accompanied by increased expression of GABAergic synaptic elements. Among GABA-A receptor subunits, β3 was the most highly expressed, while the postnatally expressed *δ* subunit was not detected, consistent with second-trimester fetal brain transcriptomic data (Cao et al., 2020). Similarly, parvalbumin-positive interneurons, typically born later in development, were absent from our cultures. These findings align with previous transcriptomic studies showing that cortical organoid development *in vitro* parallels human cortical maturation through the mid-to-late fetal periods (PCW19–24), characterized by enrichment of synaptic transmission genes and downregulation of cell cycle-related genes (Paşca et al., 2015). Building on this, by exposing organoid cultures to ALLO from DIV27 to DIV68, corresponding to the maturation phase analogous to the second trimester, we aimed to model placental ALLO exposure during mid-gestation. This design allowed us to assess ALLO’s presence at DIV55 (approximating PCW19) (Paşca et al., 2015), when placental ALLO is normally available) and ALLO withdrawal at DIV78 (approximating PCW24) (Paşca et al., 2015, or extremely premature birth, when placental support is lost, Supplementary Figure S10).

We highlight ALLO withdrawal’s role, which occurs during birth, notably preterm birth, in supporting the GABAergic system. Indeed, preterm birth and subsequent placental loss significantly increase the risk for neuropsychiatric disorders characterized by disrupted GABAergic signaling. This association is supported by multiple lines of evidence: impaired cortical GABAergic system maturation (Lacaille et al., 2021), decreased density of cortical GABAergic interneurons (Lacaille et al., 2019), reduced GABA concentrations in preterm infant prefrontal cortex (Basu et al., 2023), GABA-A receptor disruptions in schizophrenia (Nosarti et al., 2012; Bristow et al., 2015), and the prevalence of conditions like epilepsy, ASD, and attention deficits and hyperactivity disorders in preterm survivors (Schür et al., 2016; Crump et al., 2021) Complementing these observations, accumulating evidence indicates that prenatal sex steroids and placental growth factor (PlGF) are associated with autistic traits in offspring (Kratimenos and Penn, 2019; Vacher et al., 2021, 2025; Bakalar et al., 2022; Tsompanidis et al., 2023). Here, we provide a robust foundation for understanding how ALLO directly influences cortical transcriptional changes, using a consistent experimental approach, and differentiating organoids into ventral and dorsal cortical progenitors. By focusing on developmental points relevant to preterm birth, and examining both ALLO exposure and withdrawal, we offer new perspectives on ALLO’s role in shaping the cortical GABAergic system.

### Limitations and future directions

Several limitations warrant consideration and point toward important directions for future research. Our use of organoids is derived from a single cell line and limits genetic diversity, potentially not fully representing population-wide responses to ALLO. Future studies should incorporate multiple cell lines, particularly individuals with disorders involving GABAergic dysfunction such as ASD or schizophrenia (Levy and Paşca, 2023; Hali et al., 2025), and models of prematurity (Pașca et al., 2019), to address variations in neurosteroid sensitivity and enhance translational relevance. More extensive temporal protein analysis will be essential to validate our gene expression findings, characterize the cellular mechanisms involved and track cell lineage changes. More frequent sampling would provide finer resolution of the temporal dynamics, especially during immediate ALLO exposure and post-withdrawal periods. Additionally, we used commercially available culture media and serum, which may contain baseline levels of steroids. Current limitations of organoid technology include the difficulty of studying sex differences (Kelava et al., 2022; Pavlinek et al., 2024), a critical aspect for understanding neurosteroid actions in neurodevelopment. Finally, as an *in vitro* model, cortical organoids cannot replicate the systemic interactions and environmental complexity of a living organism. They lack the complex maternal-fetal interface, including interactions with other placental hormones, immune factors, and neural cell types that may modulate ALLO effects *in vivo*. This limitation highlights the need for complementary *in vivo* approaches or co-culture systems to better capture the physiological context of cortical development.

## Conclusion

In conclusion, using human organoids, we present robust evidence that ALLO is a critical regulator of cortical maturation and GABAergic system development. Our findings align with clinical observations of impaired cortical GABAergic signaling in preterm infants, who experience placental ALLO withdrawal many weeks before reaching term. Given the limited treatment options currently available for addressing neurodevelopmental risks in this population, this research paves the way for novel therapies based on placental hormone replacement. While neurosteroid-based interventions represent one potential avenue for neuroprotection in preterm infants, extensive preclinical and clinical development is still required. Our organoid model provides a platform for testing candidate interventions and understanding their mechanisms in human cortical tissue.

## Data Availability

The original contributions presented in the study are included in the article/Supplementary material, further inquiries can be directed to the corresponding authors.

## Footnotes

1
https://stemcells.nindsgenetics.org

## References

[ref1] Arlotta P. Paşca S. P. (2019). Cell diversity in the human cerebral cortex: from the embryo to brain organoids. Curr. Opin. Neurobiol. 56, 194–198. doi: 10.1016/j.conb.2019.03.001, 31051421

[ref2] Back S. A. Miller S. P. (2014). Brain injury in premature neonates: a primary cerebral dysmaturation disorder? Ann. Neurol. 75, 469–486. doi: 10.1002/ana.24132, 24615937 PMC5989572

[ref3] Bakalar D. O’Reilly J. J. Lacaille H. Salzbank J. Ellegood J. Lerch J. P. . (2022). Lack of placental neurosteroid alters cortical development and female somatosensory function. Front. Endocrinol. 13:972033. doi: 10.3389/fendo.2022.972033, 36313771 PMC9606442

[ref4] Basu S. K. Pradhan S. Sharker Y. M. Kapse K. J. Murnick J. Chang T. . (2023). Severity of prematurity and age impact early postnatal development of GABA and glutamate systems. Cereb. Cortex 33, 7386–7394. doi: 10.1093/cercor/bhad046, 36843135 PMC10267637

[ref5] Birey F. Andersen J. Makinson C. D. Islam S. Wei W. Huber N. . (2017). Assembly of functionally integrated human forebrain spheroids. Nature 545, 54–59. doi: 10.1038/nature22330, 28445465 PMC5805137

[ref6] Bristow G. C. Bostrom J. A. Haroutunian V. Sodhi M. S. (2015). Sex differences in GABAergic gene expression occur in the anterior cingulate cortex in schizophrenia. Schizophr. Res. 167, 57–63. doi: 10.1016/j.schres.2015.01.025, 25660468 PMC4524801

[ref7] Buccitelli C. Selbach M. (2020). mRNAs, proteins and the emerging principles of gene expression control. Nat. Rev. Genet. 21, 630–644. doi: 10.1038/s41576-020-0258-4, 32709985

[ref8] Cao J. O’Day D. R. Pliner H. A. Kingsley P. D. Deng M. Daza R. M. . (2020). A human cell atlas of fetal gene expression. Science 370:eaba772. doi: 10.1126/science.aba7721, 33184181 PMC7780123

[ref9] Chang Y. Huang Y. Whiteaker P. (2010). Mechanism of allosteric modulation of the Cys-loop receptors. Pharmaceuticals 3, 2592–2609. doi: 10.3390/ph3082592, 27713368 PMC4033940

[ref10] Charalampopoulos I. Tsatsanis C. Dermitzaki E. Alexaki V.-I. Castanas E. Margioris A. N. . (2004). Dehydroepiandrosterone and allopregnanolone protect sympathoadrenal medulla cells against apoptosis via antiapoptotic Bcl-2 proteins. Proc. Natl. Acad. Sci. USA 101, 8209–8214. doi: 10.1073/pnas.0306631101, 15148390 PMC419582

[ref11] Chen S. Wang T. Yao J. Brinton R. D. (2020). Allopregnanolone promotes neuronal and oligodendrocyte differentiation in vitro and in vivo: therapeutic implication for Alzheimer’s disease. Neurotherapeutics 17, 1813–1824. doi: 10.1007/s13311-020-00874-x, 32632771 PMC7851314

[ref12] Ciceri G. Baggiolini A. Cho H. S. Kshirsagar M. Benito-Kwiecinski S. Walsh R. M. . (2024). An epigenetic barrier sets the timing of human neuronal maturation. Nature 626, 881–890. doi: 10.1038/s41586-023-06984-8, 38297124 PMC10881400

[ref13] Concas A. Mostallino M. C. Porcu P. Follesa P. Barbaccia M. L. Trabucchi M. . (1998). Role of brain allopregnanolone in the plasticity of gamma-aminobutyric acid type a receptor in rat brain during pregnancy and after delivery. Proc. Natl. Acad. Sci. USA 95, 13284–13289. doi: 10.1073/pnas.95.22.13284, 9789080 PMC23784

[ref14] Courtney J.-M. Morris G. P. Cleary E. M. Howells D. W. Sutherland B. A. (2022). Automated quantification of multiple cell types in fluorescently labeled whole mouse brain sections using QuPath. Bio Protoc. 12:e4459. doi: 10.21769/BioProtoc.4459, 35937935 PMC9303822

[ref15] Crump C. Sundquist J. Sundquist K. (2021). Preterm or early term birth and risk of autism. Pediatrics 148:e2020032300. doi: 10.1542/peds.2020-032300, 34380775 PMC9809198

[ref16] de Lecea L. del Río J. A. Soriano E. (1995). Developmental expression of parvalbumin mRNA in the cerebral cortex and hippocampus of the rat. Brain Res. Mol. Brain Res. 32, 1–13. doi: 10.1016/0169-328x(95)00056-x, 7494447

[ref17] Dean J. M. Bennet L. Back S. A. McClendon E. Riddle A. Gunn A. J. (2014). What brakes the preterm brain? An arresting story. Pediatr. Res. 75, 227–233. doi: 10.1038/pr.2013.189, 24336432

[ref18] du Plessis A. J. Volpe J. J. (2025). “Chapter 10 - placental conditions with consequences for the fetal brain” in Volpe’s neurology of the newborn. ed. Volpe J. J.. 7th ed (St. Louis, MO: Elsevier), 236–262.e9.

[ref19] França L. G. S. Ciarrusta J. Gale-Grant O. Fenn-Moltu S. Fitzgibbon S. Chew A. . (2024). Neonatal brain dynamic functional connectivity in term and preterm infants and its association with early childhood neurodevelopment. Nat. Commun. 15:16. doi: 10.1038/s41467-023-44050-z, 38331941 PMC10853532

[ref20] Gago N. El-Etr M. Sananès N. Cadepond F. Samuel D. Avellana-Adalid V. . (2004). 3alpha,5alpha-Tetrahydroprogesterone (allopregnanolone) and gamma-aminobutyric acid: autocrine/paracrine interactions in the control of neonatal PSA-NCAM+ progenitor proliferation. J. Neurosci. Res. 78, 770–783. doi: 10.1002/jnr.20348, 15523635

[ref21] Gandal M. J. Nesbitt A. M. McCurdy R. M. Alter M. D. (2012). Measuring the maturity of the fast-spiking interneuron transcriptional program in autism, schizophrenia, and bipolar disorder. PLoS One 7:e41215. doi: 10.1371/journal.pone.0041215, 22936973 PMC3427326

[ref22] Gilbert Evans S. E. Ross L. E. Sellers E. M. Purdy R. H. Romach M. K. (2005). 3alpha-reduced neuroactive steroids and their precursors during pregnancy and the postpartum period. Gynecol. Endocrinol. 21, 268–279. doi: 10.1080/09513590500361747, 16373246

[ref23] Gordon A. Yoon S.-J. Tran S. S. Makinson C. D. Park J. Y. Andersen J. . (2021). Long-term maturation of human cortical organoids matches key early postnatal transitions. Nat. Neurosci. 24, 331–342. doi: 10.1038/s41593-021-00802-y, 33619405 PMC8109149

[ref24] Hali S. Yao X. Hao G. Jin Z.-L. Fu K. Li Y. . (2025). Differentiation defect into GABAergic neurons in cerebral organoids from autism patients. CNS Neurosci. Ther. 31:e70449. doi: 10.1111/cns.70449, 40457513 PMC12129711

[ref25] Hee Chung E. Chou J. Brown K. A. (2020). Neurodevelopmental outcomes of preterm infants: a recent literature review. Transl. Pediatr. 9, S3–S8. doi: 10.21037/tp.2019.09.10, 32206579 PMC7082240

[ref26] Hernandez G. D. Brinton R. D. (2022). Allopregnanolone: regenerative therapeutic to restore neurological health. Neurobiol. Stress 21:100502. doi: 10.1016/j.ynstr.2022.100502, 36532370 PMC9755066

[ref27] Hosie A. M. Clarke L. da Silva H. Smart T. G. (2009). Conserved site for neurosteroid modulation of GABA a receptors. Neuropharmacology 56, 149–154. doi: 10.1016/j.neuropharm.2008.07.050, 18762201

[ref28] Hosie A. M. Wilkins M. E. da Silva H. M. A. Smart T. G. (2006). Endogenous neurosteroids regulate GABAA receptors through two discrete transmembrane sites. Nature 444, 486–489. doi: 10.1038/nature05324, 17108970

[ref29] Kelava I. Chiaradia I. Pellegrini L. Kalinka A. T. Lancaster M. A. (2022). Androgens increase excitatory neurogenic potential in human brain organoids. Nature 602, 112–116. doi: 10.1038/s41586-021-04330-4, 35046577 PMC7612328

[ref30] Kelleher M. A. Palliser H. K. Walker D. W. Hirst J. J. (2011). Sex-dependent effect of a low neurosteroid environment and intrauterine growth restriction on foetal guinea pig brain development. J. Endocrinol. 208, 301–309. doi: 10.1677/JOE-10-0248, 21149437

[ref31] Kelley K. W. Pașca S. P. (2022). Human brain organogenesis: toward a cellular understanding of development and disease. Cell 185, 42–61. doi: 10.1016/j.cell.2021.10.003, 34774127 PMC13523617

[ref32] Kilb W. Kirischuk S. Luhmann H. J. (2013). Role of tonic GABAergic currents during pre- and early postnatal rodent development. Front. Neural Circ. 7:139. doi: 10.3389/fncir.2013.00139, 24027498 PMC3760143

[ref33] Kratimenos P. Penn A. A. (2019). Placental programming of neuropsychiatric disease. Pediatr. Res. 86, 157–164. doi: 10.1038/s41390-019-0405-9, 31003234 PMC11906117

[ref34] Kumar A. Qian M. Xu Y. Benz A. Covey D. F. Zorumski C. F. . (2025). Unravelling the multifaceted actions of neurosteroids: machine learning and in vitro screening for novel target discovery. Br. J. Pharmacol. 182, 5226–5246. doi: 10.1111/bph.70114, 40555397 PMC13531688

[ref35] Lacaille H. Vacher C.-M. Bakalar D. O’Reilly J. J. Salzbank J. Penn A. A. (2019). Impaired interneuron development in a novel model of neonatal brain injury. ENeuro 6:ENEURO.0300-18.2019. doi: 10.1523/ENEURO.0300-18.2019, 30809588 PMC6390196

[ref36] Lacaille H. Vacher C.-M. Penn A. A. (2021). Preterm birth alters the maturation of the GABAergic system in the human prefrontal cortex. Front. Mol. Neurosci. 14:827370. doi: 10.3389/fnmol.2021.827370, 35185465 PMC8852329

[ref37] Lammertink F. van den Heuvel M. P. Hermans E. J. Dudink J. Tataranno M. L. Benders M. J. N. L. . (2022). Early-life stress exposure and large-scale covariance brain networks in extremely preterm-born infants. Transl. Psychiatry 12:256. doi: 10.1038/s41398-022-02019-4, 35717524 PMC9206645

[ref38] Landeira B. S. Santana T. T. d. S. Araújo J. A. d. M. Tabet E. I. Tannous B. A. Schroeder T. . (2018). Activity-independent effects of CREB on neuronal survival and differentiation during mouse cerebral cortex development. Cereb. Cortex 28, 538–548. doi: 10.1093/cercor/bhw387 27999124 PMC6248567

[ref39] Legesse D. H. Fan C. Teng J. Zhuang Y. Howard R. J. Noviello C. M. . (2023). Structural insights into opposing actions of neurosteroids on GABA(a) receptors. Nat. Commun. 14:5091. doi: 10.1038/s41467-023-40800-1, 37607940 PMC10444788

[ref40] Levy R. J. Paşca S. P. (2023). What have organoids and Assembloids taught us about the pathophysiology of neuropsychiatric disorders? Biol. Psychiatry 93, 632–641. doi: 10.1016/j.biopsych.2022.11.017, 36739210

[ref41] Luisi S. Petraglia F. Benedetto C. Nappi R. E. Bernardi F. Fadalti M. . (2000). Serum allopregnanolone levels in pregnant women: changes during pregnancy, at delivery, and in hypertensive patients. J. Clin. Endocrinol. Metab. 85, 2429–2433. doi: 10.1210/jcem.85.7.6675, 10902789

[ref42] Mariani J. Coppola G. Zhang P. Abyzov A. Provini L. Tomasini L. . (2015). FOXG1-dependent dysregulation of GABA/glutamate neuron differentiation in autism Spectrum disorders. Cell 162, 375–390. doi: 10.1016/j.cell.2015.06.034, 26186191 PMC4519016

[ref43] Miyoshi G. Ueta Y. Natsubori A. Hiraga K. Osaki H. Yagasaki Y. . (2021). FoxG1 regulates the formation of cortical GABAergic circuit during an early postnatal critical period resulting in autism spectrum disorder-like phenotypes. Nat. Commun. 12:3773. doi: 10.1038/s41467-021-23987-z, 34145239 PMC8213811

[ref44] Miyoshi G. Ueta Y. Yagasaki Y. Kishi Y. Fishell G. Machold R. P. . (2024). Developmental trajectories of GABAergic cortical interneurons are sequentially modulated by dynamic FoxG1 expression levels. Proc. Natl. Acad. Sci. USA 121:e2317783121. doi: 10.1073/pnas.2317783121, 38588430 PMC11032493

[ref45] Nosarti C. Reichenberg A. Murray R. M. Cnattingius S. Lambe M. P. Yin L. . (2012). Preterm birth and psychiatric disorders in young adult life. Arch. Gen. Psychiatry 69, E1–E8. doi: 10.1001/archgenpsychiatry.2011.1374, 22660967

[ref46] Nueda M. J. Tarazona S. Conesa A. (2014). Next maSigPro: updating maSigPro bioconductor package for RNA-seq time series. Bioinformatics 30, 2598–2602. doi: 10.1093/bioinformatics/btu333, 24894503 PMC4155246

[ref47] Ohuma E. O. Moller A.-B. Bradley E. Chakwera S. Hussain-Alkhateeb L. Lewin A. . (2023). National, regional, and global estimates of preterm birth in 2020, with trends from 2010: a systematic analysis. Lancet 402, 1261–1271. doi: 10.1016/S0140-6736(23)00878-4, 37805217

[ref48] Pașca A. M. Park J.-Y. Shin H.-W. Qi Q. Revah O. Krasnoff R. . (2019). Human 3D cellular model of hypoxic brain injury of prematurity. Nat. Med. 25, 784–791. doi: 10.1038/s41591-019-0436-0, 31061540 PMC7020938

[ref49] Pasca A. M. Penn A. A. (2010). The placenta: the lost neuroendocrine organ. NeoReviews 11, e64–e77. doi: 10.1542/neo.11-2-e64

[ref50] Paşca A. M. Sloan S. A. Clarke L. E. Tian Y. Makinson C. D. Huber N. . (2015). Functional cortical neurons and astrocytes from human pluripotent stem cells in 3D culture. Nat. Methods 12, 671–678. doi: 10.1038/nmeth.3415, 26005811 PMC4489980

[ref51] Patel A. Garcia Diaz A. Moore J. C. Sirabella D. Corneo B. (2020). Establishment and characterization of two iPSC lines derived from healthy controls. Stem Cell Res. 47:101926. doi: 10.1016/j.scr.2020.101926, 32738631

[ref52] Pavlinek A. Adhya D. Tsompanidis A. Warrier V. Vernon A. C. Lancaster M. . (2024). Using organoids to model sex differences in the human brain. Biol. Psychiatry Glob. Open Sci. 4:100343. doi: 10.1016/j.bpsgos.2024.100343, 39092139 PMC11292257

[ref53] Penning T. M. Burczynski M. E. Jez J. M. Hung C. F. Lin H. K. Ma H. . (2000). Human 3alpha-hydroxysteroid dehydrogenase isoforms (AKR1C1-AKR1C4) of the aldo-keto reductase superfamily: functional plasticity and tissue distribution reveals roles in the inactivation and formation of male and female sex hormones. Biochem. J. 351, 67–77. doi: 10.1042/0264-6021:3510067, 10998348 PMC1221336

[ref54] Puia G. Santi M. R. Vicini S. Pritchett D. B. Purdy R. H. Paul S. M. . (1990). Neurosteroids act on recombinant human GABAA receptors. Neuron 4, 759–765. doi: 10.1016/0896-6273(90)90202-q, 2160838

[ref55] Reis F. M. Petraglia F. (2001). The placenta as a neuroendocrine organ. Front. Horm. Res. 27, 216–228. doi: 10.1159/000061028 11450428

[ref56] Rogers C. E. Lean R. E. Wheelock M. D. Smyser C. D. (2018). Aberrant structural and functional connectivity and neurodevelopmental impairment in preterm children. J. Neurodev. Disord. 10:38. doi: 10.1186/s11689-018-9253-x, 30541449 PMC6291944

[ref57] Schür R. R. Draisma L. W. R. Wijnen J. P. Boks M. P. Koevoets M. G. J. C. Joëls M. . (2016). Brain GABA levels across psychiatric disorders: a systematic literature review and meta-analysis of (1) H-MRS studies. Hum. Brain Mapp. 37, 3337–3352. doi: 10.1002/hbm.23244, 27145016 PMC6867515

[ref58] Shimizu H. Ishizuka Y. Yamazaki H. Shirao T. (2015). Allopregnanolone increases mature excitatory synapses along dendrites via protein kinase a signaling. Neuroscience 305, 139–145. doi: 10.1016/j.neuroscience.2015.07.079, 26241343

[ref59] Sloan S. A. Andersen J. Pașca A. M. Birey F. Pașca S. P. (2018). Generation and assembly of human brain region-specific three-dimensional cultures. Nat. Protoc. 13, 2062–2085. doi: 10.1038/s41596-018-0032-7, 30202107 PMC6597009

[ref60] Smith S. S. Shen H. Gong Q. H. Zhou X. (2007). Neurosteroid regulation of GABA(a) receptors: focus on the alpha4 and delta subunits. Pharmacol. Ther. 116, 58–76. doi: 10.1016/j.pharmthera.2007.03.008, 17512983 PMC2657726

[ref61] Song I. G. (2023). Neurodevelopmental outcomes of preterm infants. Clin. Exp. Pediatr. 66, 281–287. doi: 10.3345/cep.2022.00822, 36596743 PMC10331553

[ref62] Sugasawa Y. Cheng W. W. Bracamontes J. R. Chen Z.-W. Wang L. Germann A. L. . (2020). Site-specific effects of neurosteroids on GABA(a) receptor activation and desensitization. eLife 9:e55331, 1–32. doi: 10.7554/eLife.55331, 32955433 PMC7532004

[ref63] Tsompanidis A. Warrier V. Baron-Cohen S. (2023). The genetics of autism and steroid-related traits in prenatal and postnatal life. Front. Endocrinol. 14:1126036. doi: 10.3389/fendo.2023.1126036, 37223033 PMC10200920

[ref64] Vacher C.-M. Bonnin A. Mir I. N. Penn A. A. (2023). Editorial: advances and perspectives in neuroplacentology. Front. Endocrinol. 14:1206072. doi: 10.3389/fendo.2023.1206072, 37274324 PMC10236794

[ref65] Vacher C.-M. Lacaille H. O’Reilly J. J. Salzbank J. Bakalar D. Sebaoui S. . (2021). Placental endocrine function shapes cerebellar development and social behavior. Nat. Neurosci. 24, 1392–1401. doi: 10.1038/s41593-021-00896-4, 34400844 PMC8481124

[ref66] Vacher C.-M. Tsompanidis A. Firestein M. R. Penn A. A. (2025). Neuroactive steroid exposure impacts neurodevelopment: comparison of human and rodent placental contribution. J. Neuroendocrinol. 37:e13489. doi: 10.1111/jne.13489, 39789736 PMC12213033

[ref67] Volpe J. J. (2019). Dysmaturation of premature brain: importance, cellular mechanisms, and potential interventions. Pediatr. Neurol. 95, 42–66. doi: 10.1016/j.pediatrneurol.2019.02.016, 30975474

[ref68] Wang T. Chen S. Mao Z. Shang Y. Brinton R. D. (2023). Allopregnanolone pleiotropic action in neurons and astrocytes: calcium signaling as a unifying mechanism. Front. Endocrinol. 14:1286931. doi: 10.3389/fendo.2023.1286931, 38189047 PMC10771836

[ref69] Wang J. M. Johnston P. B. Ball B. G. Brinton R. D. (2005). The neurosteroid allopregnanolone promotes proliferation of rodent and human neural progenitor cells and regulates cell-cycle gene and protein expression. J. Neurosci. 25, 4706–4718. doi: 10.1523/JNEUROSCI.4520-04.2005, 15888646 PMC6724768

[ref70] Witteveen I. F. McCoy E. Holsworth T. D. Shen C. Z. Chang W. Nance M. G. . (2023). Preterm birth accelerates the maturation of spontaneous and resting activity in the visual cortex. Front. Integr. Neurosci. 17:1149159. doi: 10.3389/fnint.2023.1149159, 37255843 PMC10225509

[ref71] Zamora-Sánchez C. J. Bello-Alvarez C. Rodríguez-Dorantes M. Camacho-Arroyo I. (2022). Allopregnanolone promotes migration and invasion of human glioblastoma cells through the protein tyrosine kinase c-Src activation. Int. J. Mol. Sci. 23:4996. doi: 10.3390/ijms23094996, 35563388 PMC9105169

[ref72] Zheleznova N. N. Sedelnikova A. Weiss D. S. (2009). Function and modulation of delta-containing GABA(a) receptors. Psychoneuroendocrinology 34, S67–S73. doi: 10.1016/j.psyneuen.2009.08.010 19766404 PMC2794972

