# Supplementary material for: Neurosteroid withdrawal disrupts GABAergic system development in human cortical organoids: implications for preterm birth
Source: Front Cell Neurosci. 2025 Dec 17;19:1715823. doi: 10.3389/fncel.2025.1715823 (PMC12753414; doi:10.3389/fncel.2025.1715823)
Supplement: Supplementary file 1 [file Data_Sheet_1.pdf]

Table S1

Gene name	Reverse	Forward
AKR1C2	CAGTGGATCTCTGTGCCACATG	CTGGTTGCAGACAGGCTTGTAC
AKR1C3	CCGAAGCAAGATTGCAGATGGC	GTGAGTTTTCCAAGGCTGGTCG
ASCL1	TCTCATCCTACTCGTCGGACGA	CTGCTTCCAAAGTCCATTCGCAC
CALB2	GATCCTGCCAACCGAAGAGAAC	CGATGTAGCCACTCCTGTCTGT
CUX1	TCCGTAGCATCCAAGGCAGACA	CTTCATCAGAGCCAGTCTCCGA
DCX	TATGCGCCGAAGCAAGTCTCCA	CATCCAAGGACAGAGGCAGGTA
DLG4	TCCACTCTGACAGTGAGACCGA	CGTCACTGTCTCGTAGCTCAGA
ENO2	CTGTATCGCCACATTGCTCAGC	AGCTTGTTGCCAGCATGAGAGC
FOXG1	AACCTGTGTTGCGCAAATGC	AAACACGGGCATATGACCAC
FOXP2	TGGATGACCGAAGCACTGCTCA	TGGGAGATGGTTTGGGCTCTGA
GABARAP	ATCTCCGAGCTGAGGATGCCTT	GACACTTTCGTCACTGTAGGCAA
GABBR1	CCTGAACAAGACATCTGGAGGAG	GCTGGCATCAAACACCACATGG
GABBR2	GTTGCTCAAGCACTACCAGTGG	TCCTCGCCATACAGAACTCCAG
GABRA1	CACAAGTCTCCTTCTGGCTCAAC	GGAGTTTCTGGCACTGATGCTC
GABRA2	CCCAATGCACTTGGAGGATTTCC	AGAGCCATCAGGAGCAACCTGT
GABRA3	TGGAAGTGGCACAGGATGGTTC	CGCTTGAGATGGAAGTGGGTTG
GABRA5	CTGCTCTACACCATGCGCTTGA	GAGCCGTTGGTCCAGACGTAAA
GABRB1	TGGCTCTGCTGGAGTATGCCTT	GCTGAGGAGAATGTTACCGTGG
GABRB3	CAGCCAAGGCAAAGAATGACCG	ATGCCGCCTGAGACCTCATTCA
GABRG1	CCTTTTCTTCTGCGGAGTCAA	CATCTGCCTTATCAACACAGTTTCC
GABRG2	GCACACTCATTGTCGTCCTATCC	CAATGGTGCTGAGGGTGGTCAT
GABRG3	AACCAACCACCACGAAGAAGA	CCTCATGTCCAGGAGGGAAT
GAD1	TGTCCAGGAAGCACCGCCATAA	TCCTTGACGAGAATGGCAGAGC
GAD2	GCCAACTCTGTGACGTGGAATC	GCTGAAAGAGGTAGGAGGCATG
GAPDH	GTCTCCTCTGACTTCAACAGCG	ACCACCCTGTTGCTGTAGCCAA
GFAP	CTGGAGAGGAAGATTGAGTCGC	ACGTCAAGCTCCACATGGACCT
GPHN	CTACACCAGTCCTGCTGTTGTC	CCACGAGAAATGATGGAGTCTGG
KCNJ3	GATCTCCATGAGGGACGGAAAAC	GAAGGAACTCACCCTCAGGTGT
LHX6	CGCATCCACTACGACACCATGA	GCTTGGGTTGACTGTCCTGTTC
MAP2	AGGCTGTAGCAGTCCTGAAAGG	CTTCCTCCACTGTGACAGTCTG
NCAM1	CATCACCTGGAGGACTTCTACC	CAGTGTACTGGATGCTCTTCAGG
NEFL	CCAAGACCTCCTCAACGTGAAG	ATGCTTCCCACGCTGGTGAAAC
NEFM	ACAACCACGACCTCAGCAGCTA	GTTGAGGAGGTCCTGGTATTCG
NES	TCAAGATGTCCCTCAGCCTGGA	AAGCTGAGGGAAGTCTTGGAGC
NR2F2	TGCACGTTGACTCAGCCGAGTA	AAGCACACTGAGACTTTTCCTGC
PAX6	CTGAGGAATCAGAGAAGACAGGC	ATGGAGCCAGATGTGAAGGAGG
PCNA	CAAGTAATGTCGATAAAGAGGAGG	GTGTCACCGTTGAAGAGAGTGG
PVALB	CTGATGGCTGCTGGAGACAAAG	GAGATTGGGTGTTCAGGGCAGA
RBFOX3	TACGCAGCCTACAGATACGCTC	TGGTTCCAATGCTGTAGGTCGC
SATB2	CAAGAGTGGCATTCAACCGCAC	ATCTCGCTCCACTTCTGGCAGA
SCL1A3	GGTTGCTGCAAGCACTCATCAC	CACGCCATTGTTCTCTTCCAGG
SLC17A7	GCAAGTACATCGAGGACGCCAT	GCCACGATGATGGCATAGACTG
SLC32A1	CTGGAACGTGACCAACGCCATC	TCATTCTCCTCGTACAGGCACG
SLC6A1	AACACAGACCGCTGCTTCTCCA	AGCGGATCTGACCTGGCTTATC
SLC6A11	GGTAGATGCTGGAACGCAGATC	GTTCAGGCAACAGAGCATGATGC
SOX10	ATGAACGCCTTCATGGTGTGGG	CGCTTGTCACTTTCGTTCAGCAG
SOX2	GCTACAGCATGATGCAGGACCA	TCTGCGAGCTGGTCATGGAGTT
SST	CCAGACTCCGTCAGTTTCTGCA	TTCCAGGGCATCATTCTCCGTC
SYP	TCGGCTTTGTGAAGGTGCTGCA	TCACTCTCGGTCTTGTTGGCAC
TTF1	CAGGACACCATGAGGAACAGCG	GCCATGTTCTTGCTCACGTCCC
TUBB3	TCAGCGTCTACTACAACGAGGC	GCCTGAAGAGATGTCCAAAGGC
VIM	AGGCAAAGCAGGAGTCCACTGA	ATCTGGCGTTCCAGGGACTCAT

Table S1: List of qPCR Reverse and Forward Human primer pairs

Supplementary methods

Figure S1

Example calculation of the developmental index between DIV78-DIV95 in VCOs

The dataset was first segmented to include two developmental time points of interest, DIV78 and DIV95 in VCOs, in this example.

For each gene in the segmented dataset, we computed the Pearson correlation between gene expression and time using only control (DMSO-treated) organoids. Genes with a significant positive correlation ($p < 0.05$) were classified as upregulated (red), while those with a significant negative correlation were classified as downregulated (green). Genes not meeting these criteria were considered non-responsive (**Panel A**). To ensure equal weighting across genes, expression values were normalized to a 0-1 scale for each gene independently over the whole-time course. **Panel B** displays an example of the scaling for GFAP (up, red) and NCAM1 (down, green).

The maturation index was calculated for each sample, including those in the ALLO-treated group, using the same set of upregulated and downregulated genes identified from the control group.

- If both gene sets were present, the index was defined as the ratio of the mean scaled expression of upregulated genes to that of downregulated genes.
- If only upregulated genes were detected, the index was defined as their mean scaled expression. The resulting index values were further normalized across samples to a 0–1 scale (**Panel C**).

Statistical comparisons between treatment groups and time points were performed using ANCOVA to assess differences in expression trajectories. Visualization included scatter plots with linear regression lines and annotated ANCOVA p-values (**Panel D**).

A

gene	cor	p.value	delta
ENO2	0.6989432	0.012231	up
GFAP	0.8039582	0.0438814	up
NCAM1	-0.950767	0.0035762	down
NEFL	0.6093312	0.0199121	up
NEFM	0.7811497	0.0466022	up
NES	-0.989739	0.0001574	down
NR2F2	-0.8403269	0.0362078	down
TTF1	0.9390645	0.0054566	up
VIM	-0.7179128	0.0410814	down

B

day	treatment	GFAP	NCAM1
d78	ALLO	0.095807	0.206594
d78	ALLO	0.020172	0.404229
d78	ALLO	0.013486	0.159078
d78	DMSO	0.230443	0.761739
d78	DMSO	0.010759	0.725256
d78	DMSO	0.024374	0.695858
d95	ALLO	0.036002	0.080034
d95	ALLO	0.150999	0.777925
d95	ALLO	0.113698	0.864101
d95	DMSO	0.460707	0.376585
d95	DMSO	0.338802	0.506739
d95	DMSO	0.240358	0.353292

C

day	treatment	up	down	mat_index_ not_scaled	mat_index
d78	ALLO	0.23221	0.266839	0.8702252	0.224743
d78	ALLO	0.087302	0.326444	0.2674346	0.008696
d78	ALLO	0.062963	0.258922	0.2431721	0
d78	DMSO	0.272712	0.343807	0.7932135	0.197141
d78	DMSO	0.111831	0.343869	0.3252131	0.029404
d78	DMSO	0.090322	0.332577	0.2715823	0.010183
d95	ALLO	0.131354	0.121657	1.0797157	0.299827
d95	ALLO	0.251413	0.293786	0.8557698	0.219562
d95	ALLO	0.208785	0.340273	0.6135828	0.132759
d95	DMSO	0.507251	0.16723	3.0332605	1
d95	DMSO	0.579906	0.220636	2.6283413	0.854872
d95	DMSO	0.301965	0.15007	2.0121608	0.634026

D

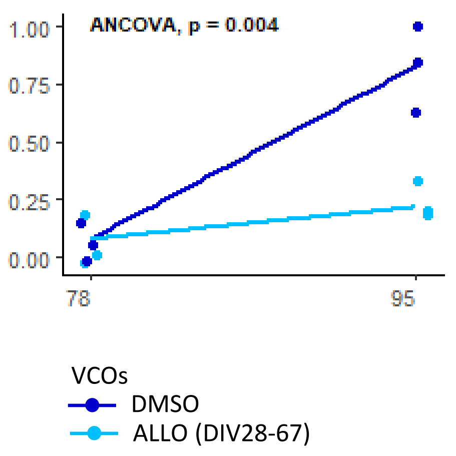

Supplementary methods

<u>Example of NeuN+ Cell Quantification in Cortical Organoids Using QuPath (v0.4.3)</u>

The whole organoid was manually outlined to define analysis regions (**Panel A**). DAPI+ nuclei were detected using the Cell Detection tool with parameters: 7 µm background radius, 5 µm cell expansion, and 20 µm intensity threshold (**Panels B-C**). For each marker (e.g., NeuN), a Random Trees classifier was trained using at least 20 manually labeled NeuN+ and NeuN− cells. The classifier utilized all available NeuN channel measurements to distinguish positive from negative cells and was applied to all detected nuclei for automated classification (**Panels D-E**). Results were reviewed for accuracy, and cell density was normalized as percentage of DAPI+ cells.

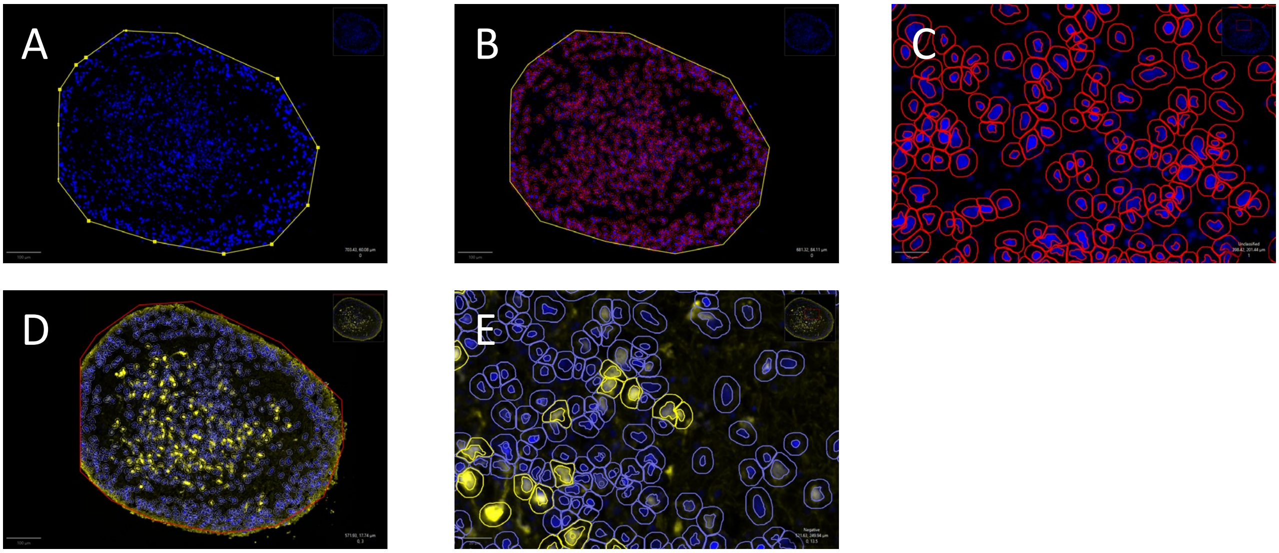

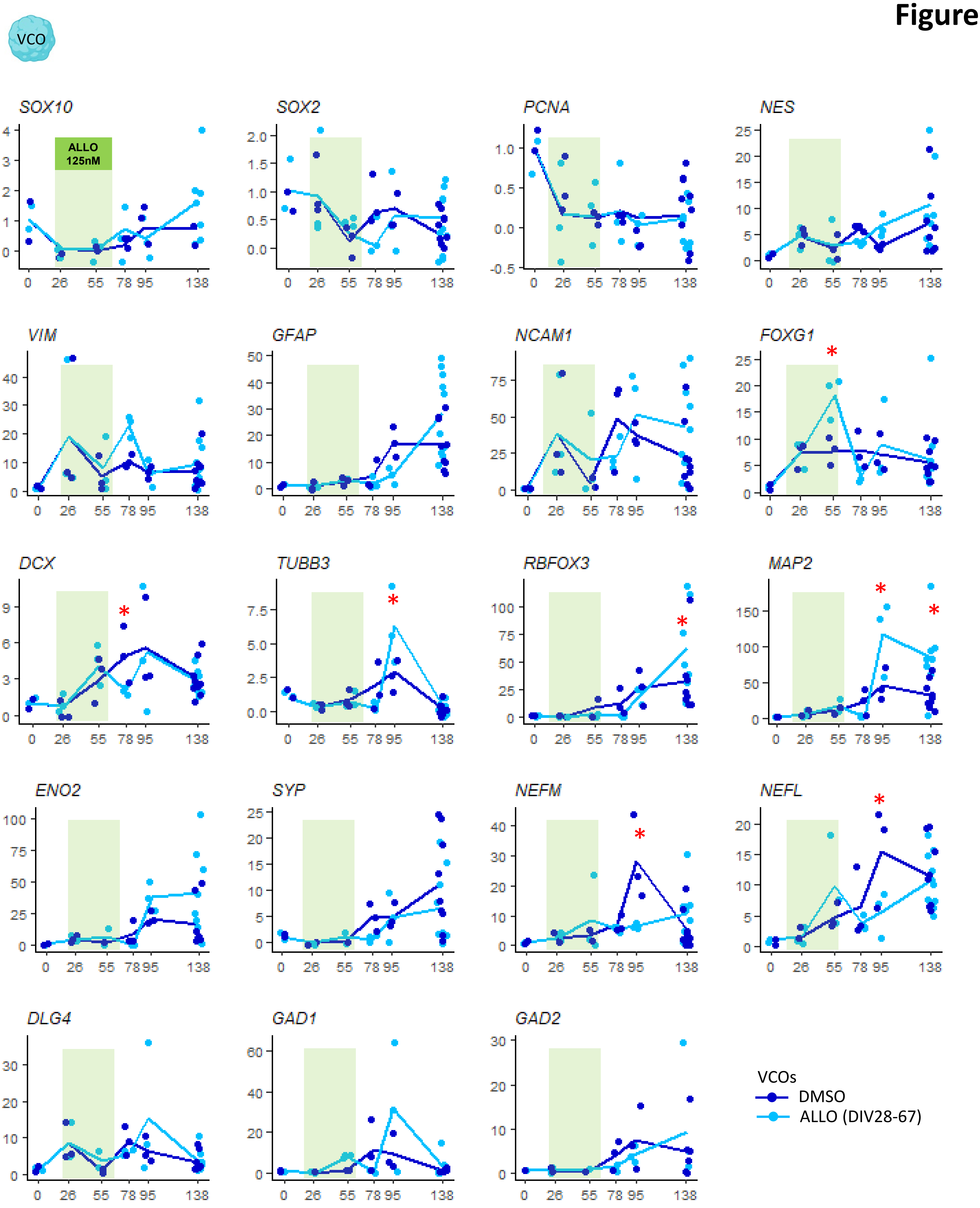

Figure S3: Transcriptional profile of neural cell development in VCOs (ventral cortical organoids) treated with DMSO (dark) or ALLO (125nM, light, from DIV28 to 67, highlighted in green) at each time point (DIV0, 26, 55, 78, 95, 138). DIV0-DIV95 n=3, DIV138 n=3-9. Two-way ANOVAs with BH correction compared treatment and day. Multiple comparisons: *p < 0.05.

Figure S4

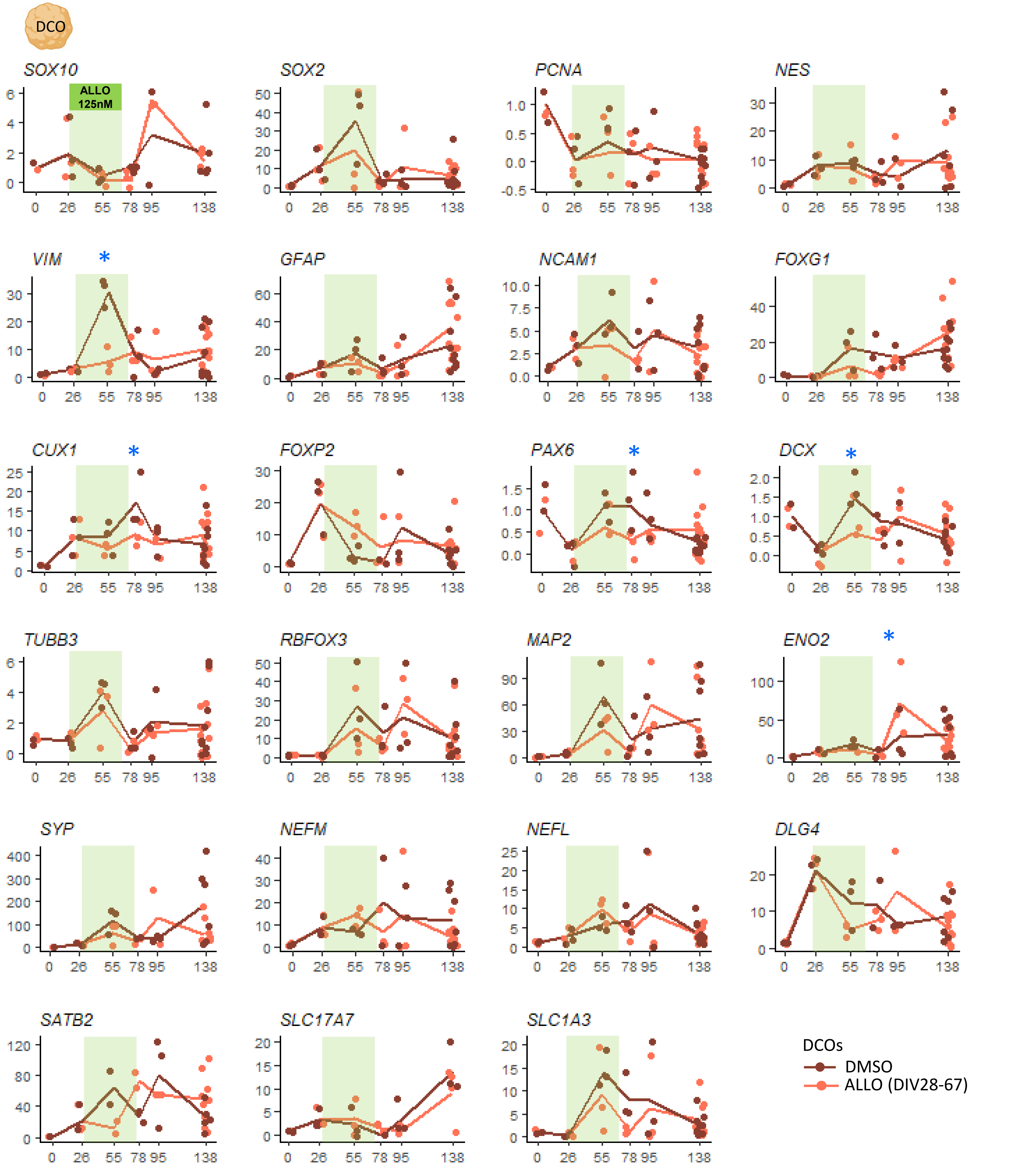

Figure S4: Transcriptional profile of neural cell development in DCOs (dorsal cortical organoids) treated with DMSO (dark) or ALLO (125nM, light, from DIV28 to 67, highlighted in green) at each time point (DIV0, 26, 55, 78, 95, 138). DIV0-DIV95 n=3, DIV138 n=3-9. Two-way ANOVAs with BH correction compared treatment and day. Multiple comparisons: *p < 0.05.

Table S2

	VCOs		DCOs	
	up-regulated genes	down-regulated genes	up-regulated genes	down-regulated genes
DIV 26 - 55	DCX, GAD1, NEFL, SLC1A3, MAP2	NA	DCX, ENO2, FOXG1, MAP2, NCAM1, NEFL, NEFM, PAX6, PCNA, RBFOX3, SATB2, SLC1A3	DLG4, FOXP2
DIV 55 - 78	ASCL1, DLG4, NCAM1, NES, NR2F2, SOX2	NA	CUX1	ENO2, MAP2, NES, PCNA, SOX2, TUBB3, VIM
DIV 78 -95	ENO2, GFAP, NEFL, NEFM, TTF1	NCAM1, NES, NR2F2, VIM	ENO2, SATB2, TUBB3	CUX1, DLG4, PAX6, VIM
DIV 95 -138	SST	DCX, DLG4, GAD1, NEFM, SOX2, TUBB3	FOXG1, NES, SLC17A7, SYP, VIM	DCX, FOXP2, NEFL, PAX6, PCNA, RBFOX3, SATB2, SLC1A3

Table S2: List of upregulated and downregulated genes in DMSO-treated ventral (VCOs) and dorsal (DCOs) cortical organoids used to calculate the segmented maturation index. Gene classification was performed separately for each time segment: DIV26–DIV55, DIV55–DIV78, DIV78–DIV95, and DIV95–DIV138.

Figure S5

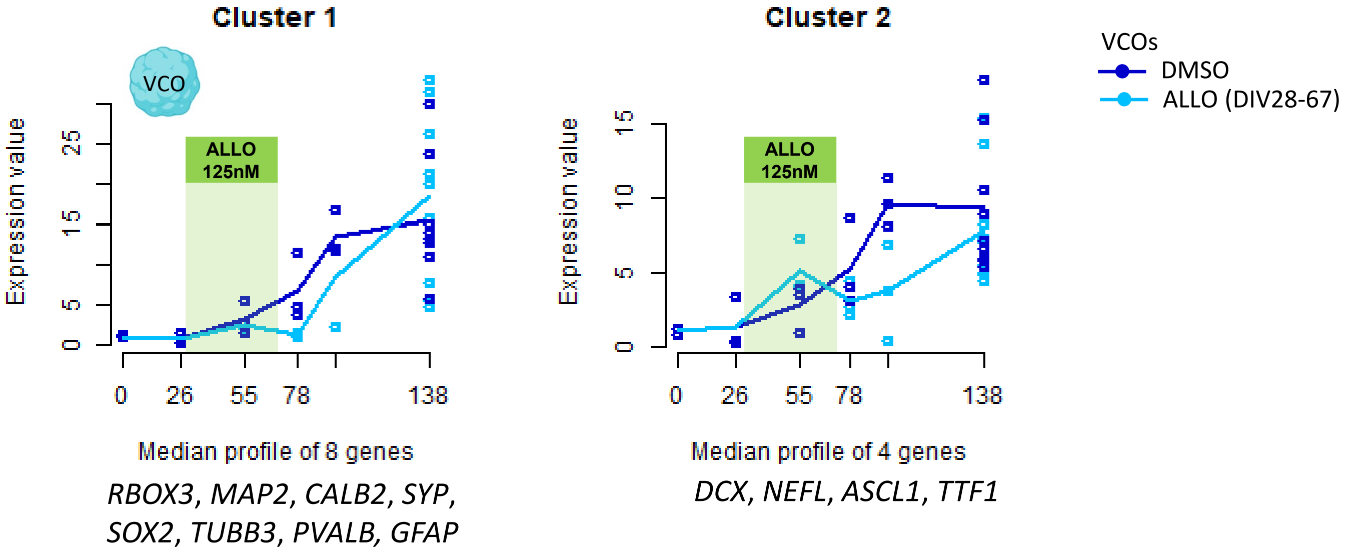

Figure S5: Global transcriptional profile of neural cell development in VCOs (ventral cortical organoids) treated with DMSO (dark) or ALLO (125nM, light, from DIV28 to 67) at each time point (DIV0, 26, 55, 78, 95, 138) clustered by their transcriptional profile across all time points (DIV0 to DIV138) using maSigPro R package. Each line represents the median expression profile for a given cluster. DIV0-DIV95 n=3, DIV138 n=6-9.

Figure S6

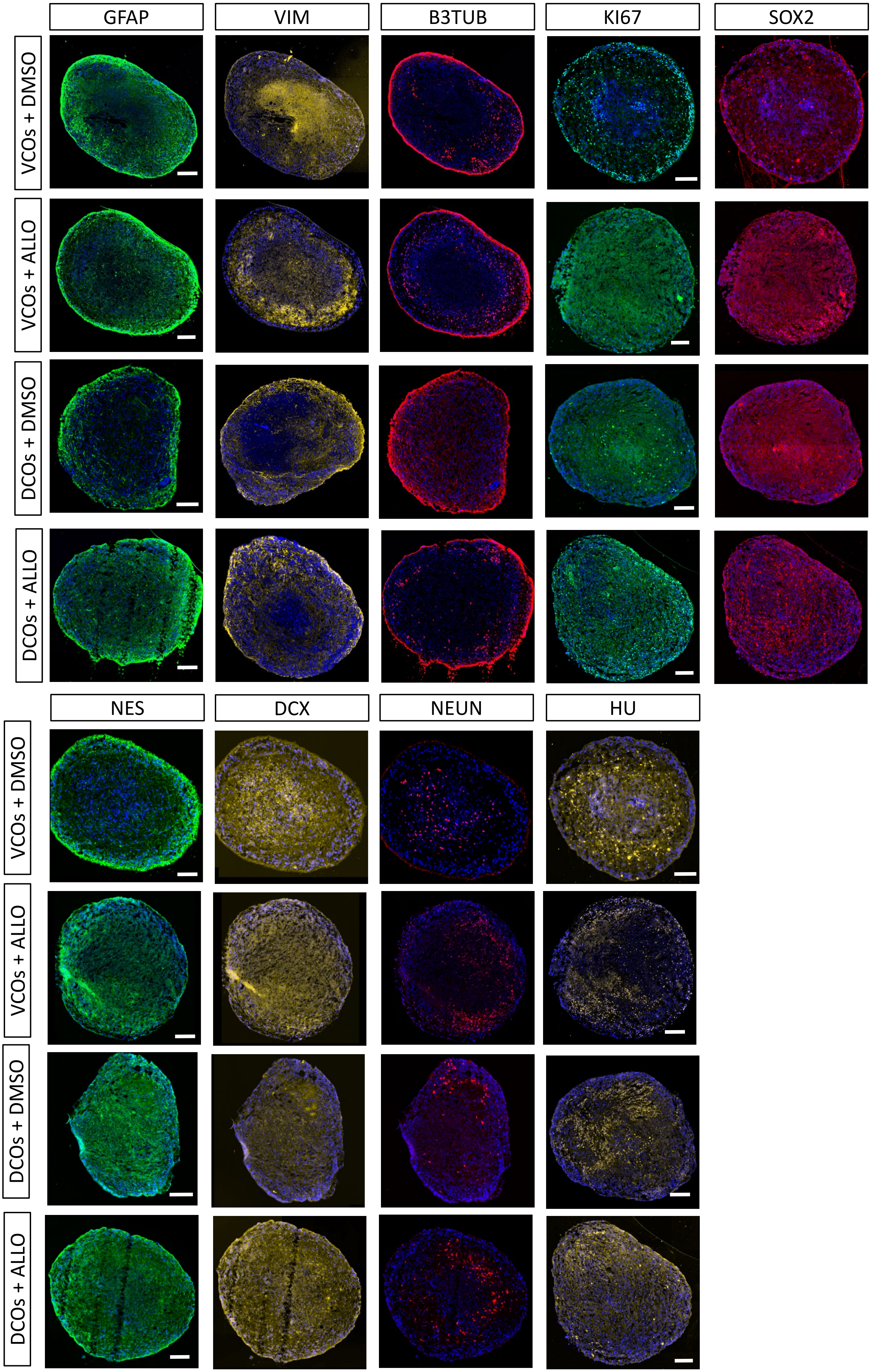
GFAP
VIM
B3TUB
KI67
SOX2
VCOs + DMSO
VCOs + ALLO
DCOs + DMSO
DCOs + ALLO
NES
DCX
NEUN
HU

Figure S6

Figure S6: Representative images of markers of neuronal development in VCOs (ventral cortical forebrain organoids) and DCOs (dorsal cortical organoids) treated with DMSO or ALLO (from DIV28 to 67). GFAP, Glial fibrillary acidic protein; VIM, Vimentin; B3TUB, Beta III Tubulin; Ki67; SRY-box 2, SOX2; NES, nestin; DCX, Doublecortin; NEUN and HU. Scale = 100µm.

Figure S7

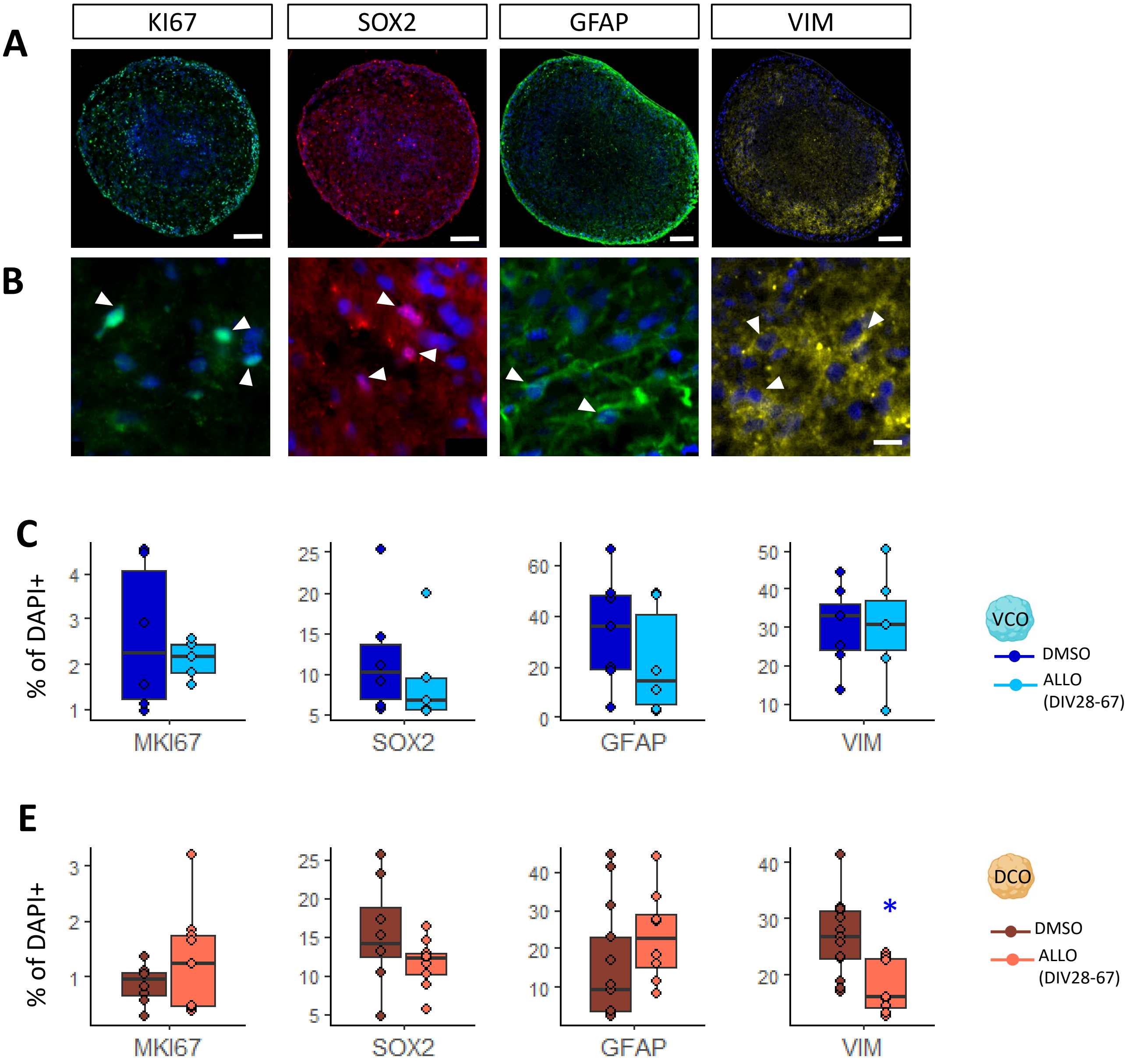

Figure S7: Effect of ALLO on additional makers of neuronal development. Cumulative effect of ALLO exposure and withdrawal at DIV138. **A** Representative illustration of whole organoid staining, scale = 100µm. **B** Representative high magnification illustrations of positive cells, scale = 10µm, arrows. **C-D** Quantification in **C** VCOs (ventral cortical organoids, blue) and **D** DCOs (dorsal cortical organoids, orange) treated with DMSO (dark) or ALLO (125nM, light, from DIV28 to 67), normalized as percentage of MKi67; SRY-box 2, SOX2; GFAP, Glial fibrillary acidic protein; VIM, Vimentin. n=7-9. Two-way ANOVAs with BH correction compared treatments and markers. Multiple comparisons: *$p < 0.05$.

Figure S8

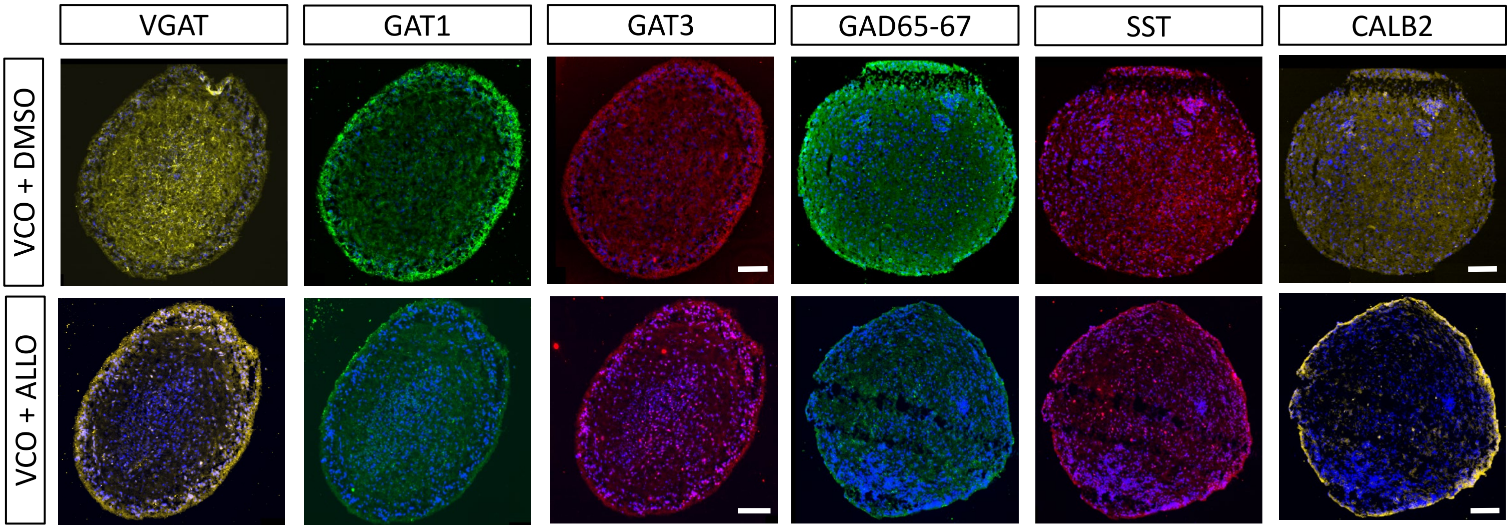

Figure S8: Representative images GABAergic markers in VCOs (ventral cortical forebrain organoids) treated with DMSO or ALLO (from DIV28 to 67). VGAT, Vesicular GABA transporter; GAT1, GABA transporter 1; GAT3, GABA transporter 3; GAD65-67, Glutamate Decarboxylase 65-67; SST, Somatostatin; CALB2, Calretinin. Scale = 100µm.

Figure S9

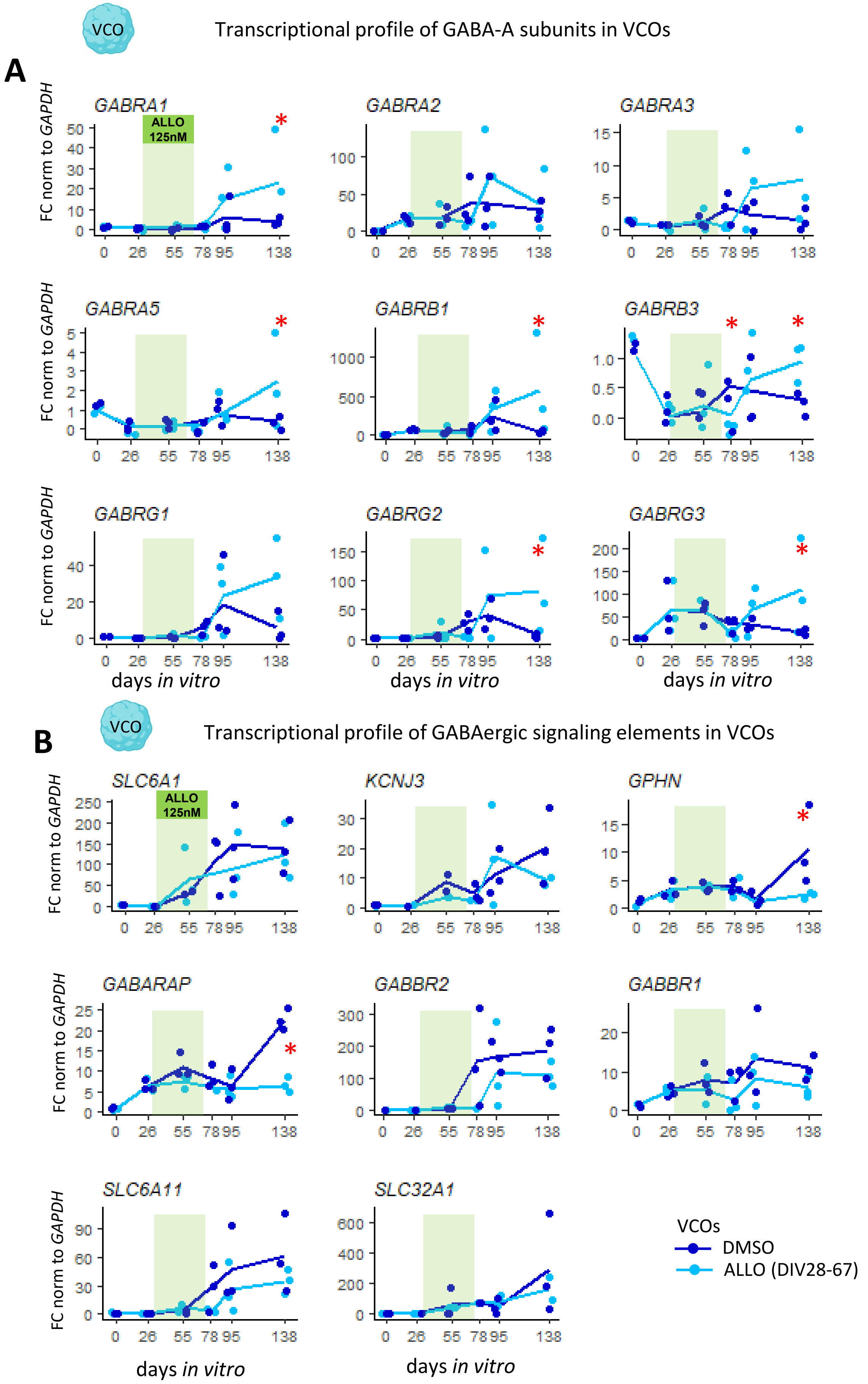

Figure S9: Transcriptional profile of GABA-related elements. A Transcriptional profile of GABA-A subunits in VCOs (ventral cortical organoids) treated with DMSO (dark) or ALLO (125nM, light, from DIV28 to 67, highlighted in green) at each time point (DIV0, 26, 55, 78, 95, 138). **B** Transcriptional profile of GABAergic signaling elements in VCOs. n=3. Two-way ANOVAs with BH correction compared treatment and day. Multiple comparisons: *p < 0.05.

Figure S10

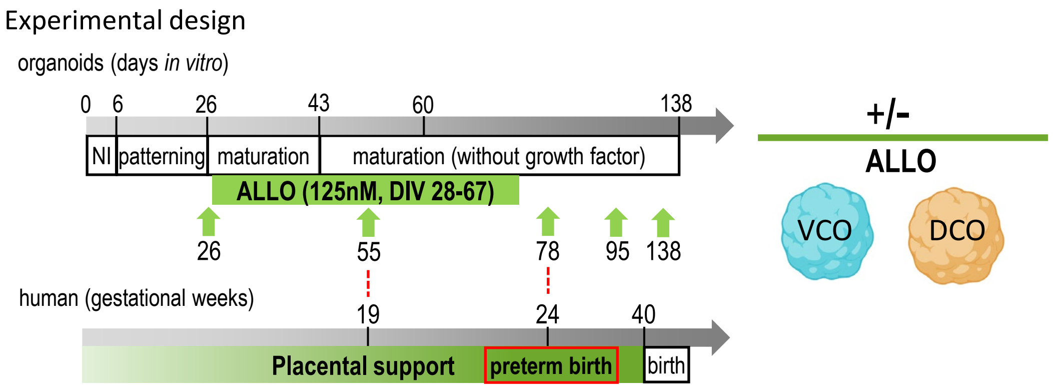

Figure S10: Schematic of experimental design and parallels with human gestational weeks.
